# STOP1 Regulates *LKS1* Transcription and Coordinates K^+^/NH_4_^+^ Balance in *Arabidopsis* Response to Low-K^+^ Stress

**DOI:** 10.3390/ijms23010383

**Published:** 2021-12-29

**Authors:** Zhi-Fang Wang, Ting-Wei Mi, Yong-Qiang Gao, Han-Qian Feng, Wei-Hua Wu, Yi Wang

**Affiliations:** State Key Laboratory of Plant Physiology and Biochemistry (SKLPPB), College of Biological Sciences, China Agricultural University, Beijing 100193, China; cctvwzf@126.com (Z.-F.W.); mitingwei@163.com (T.-W.M.); gyq20071021@163.com (Y.-Q.G.); fenghq@zaas.ac.cn (H.-Q.F.); whwu@cau.edu.cn (W.-H.W.)

**Keywords:** low K^+^, high NH_4_^+^, STOP1, LKS1/CIPK23, transcriptional regulation

## Abstract

Potassium and nitrogen are essential mineral elements for plant growth and development. The protein kinase LKS1/CIPK23 is involved in both K^+^ and NH_4_^+^ uptake in *Arabidopsis* root. The transcripts of *LKS1* can be induced by low K^+^ (0.1 mM) and high NH_4_^+^ (30 mM); however, the molecular mechanism is still unknown. In this study, we isolated the transcription factor STOP1 that positively regulates *LKS1* transcription in *Arabidopsis* responses to both low-K^+^ and high-NH_4_^+^ stresses. STOP1 proteins can directly bind to the *LKS1* promoter, promoting its transcription. The *stop1* mutants displayed a leaf chlorosis phenotype similar to *lks1* mutant when grown on low-K^+^ and high-NH_4_^+^ medium. On the other hand, *STOP1* overexpressing plants exhibited a similar tolerant phenotype to *LKS1* overexpressing plants. The transcript level of *STOP1* was only upregulated by low K^+^ rather than high NH_4_^+^; however, the accumulation of STOP1 protein in the nucleus was required for the upregulation of *LKS1* transcripts in both low-K^+^ and high-NH_4_^+^ responses. Our data demonstrate that STOP1 positively regulates *LKS1* transcription under low-K^+^ and high-NH_4_^+^ conditions; therefore, LKS1 promotes K^+^ uptake and inhibits NH_4_^+^ uptake. The STOP1/LKS1 pathway plays crucial roles in K^+^ and NH_4_^+^ homeostasis, which coordinates potassium and nitrogen balance in plants in response to external fluctuating nutrient levels.

## 1. Introduction

As one of the most abundant nutrient ions, potassium (K^+^) plays crucial roles in plant growth and development, including enzyme activation, osmotic regulation, electrical neutralization, and membrane potential maintenance [1,2,3,4]. However, the available K^+^ concentration in soil is variable and relatively low for plant growth [4,5]. Therefore, plants often suffer low-K^+^ stress in the natural environment. Under K^+^-limited conditions, plants show leaf chlorosis phenotype, starting with older leaves, a typical K^+^-deficient symptom; subsequently, plant growth and development are inhibited [6,7]. In agricultural production, K^+^ deficiency will significantly reduce crop yield and quality [3,8]. Therefore, investigation of the mechanisms underlying how plants respond to low-K^+^ stress will provide important insights into plant KUE (K utilization efficiency) improvement.

Plants absorb K^+^ from the environment through a series of K^+^ channels and transporters. In *Arabidopsis*, the Shaker family inward K^+^ channel AKT1 (*ARABIDOPSIS* K^+^ TRANSPORTER 1) and the KUP/HAK/KT family K^+^ transporter HAK5 (HIGH-AFFINITY K^+^ TRANSPORTER 5) are considered the most important components for root K^+^ uptake [9,10,11,12,13]. Their homologs in crops are also responsible for K^+^ uptake, and they determine crop yield and stress resistance [14,15,16]. NH_4_^+^ is an important nitrogen source for plants. NH_4_^+^ and K^+^ display many similarities, such as hydrated diameters, charge, and influence on membrane potentials, which result in an antagonism between the two ions [17,18]. External NH_4_^+^ inhibits HAK5-mediated high-affinity K^+^ uptake, as well as represses the upregulation of the *HAK5* transcript under low K^+^ conditions [10,19,20]. By contrast, AKT1-mediated K^+^ uptake is not affected by NH_4_^+^ [10].

Our previous study identified an AKT1-mediated K^+^ uptake pathway in *Arabidopsis*. The protein kinase LKS1/CIPK23 (LOW POTASSIUM SENSITIVITY 1/CBL-INTERACTING PROTEIN KINASE 23), interacting with the Ca^2+^-binding proteins CBL1/CBL9, phosphorylates AKT1 to enhance K^+^ uptake under low-K^+^ stress [11]. A similar regulatory pathway was also identified in rice [14]. A recent study revealed that the CBL1/9-CIPK23 complex also phosphorylates HAK5 to promote high-affinity K^+^ uptake under low-K^+^ conditions [21,22]. Furthermore, the CBL1/9–CIPK23 complex can also regulate nitrate (NO_3_^−^) and ammonium (NH_4_^+^) uptake in *Arabidopsis* root [23,24]. These studies demonstrate that LKS1/CIPK23 is essential for K^+^/NO_3_^−^/NH_4_^+^ uptake and ion homeostasis under nutrient deficient conditions. However, how is LKS1/CIPK23 regulated? Low-K^+^ stress can strongly induce *LKS1* transcripts in *Arabidopsis* root, which is one of most important mechanisms in the plant response to K^+^ deficiency [11]. In addition, high NH_4_^+^ upregulates *LKS1/CIPK23* transcripts, suggesting that the transcriptional regulation of *LKS1* is crucial for plants in response to both low K^+^ and high NH_4_^+^ to coordinate K^+^/NH_4_^+^ ion homeostasis. However, the transcriptional mechanism is little understood.

STOP1 (SENSITIVE TO PROTON RHIZOTOXICITY 1), a C2H2 zinc finger protein, functions as a crucial transcription factor that is involved in the *Arabidopsis* response to low-pH and -aluminum (Al^3+^) stresses by regulating the transcription of malate transporter *ALMT1* (ALUMINUM-ACTIVATED MALATE TRANSPORTER 1) and citrate transporter *MATE* (MULTIDRUG AND TOXIC COMPOUND EXTRUSION) [25,26,27]. Recent studies revealed that STOP1 can also participate in the regulation of root cell elongation under low Pi stress [28,29]. A growing number of studies have indicated that the STOP1-like proteins from rice (*Oryza sativa*) [30], *Eucalyptus* [31], buckwheat (*Fagopyrum esculentum* Moench) [32], rice bean (*Vigna umbellata*) [33], and tobacco (*Nicotiana tabacum*) [34] show similar functions in low-pH and -aluminum (Al^3+^) tolerance. These homologs are much conserved and regulate similar downstream target genes in different plant species [31,32,33,34,35]).

In the present study, using a high-throughput screening method, we identified the transcription factor STOP1 that positively regulates *LKS1* transcription in *Arabidopsis* responses to both low-K^+^ and high-NH_4_^+^ stresses. The *STOP1* transcript level is upregulated by low K^+^ but not high NH_4_^+^. STOP1 proteins accumulate in the nucleus under low-K^+^ or high-NH_4_^+^ conditions, and they directly bind to the *LKS1* promoter to upregulate *LKS1* transcription. Subsequently, LKS1 phosphorylates AKT1 and HAK5 to enhance K^+^ uptake, while it phosphorylates AMTs (HIGH-AFFINITY AMMONIUM TRANSPORTERS) to inhibit NH_4_^+^ uptake. The *STOP1/LKS1* pathway plays crucial roles in regulating K^+^/NH_4_^+^ ion homeostasis to strengthen *Arabidopsis* tolerance to low-K^+^ stress.

## 2. Results

### 2.1. High-Throughput Screening of the Transcription Factors of LKS1 Gene

**Figure 1:**
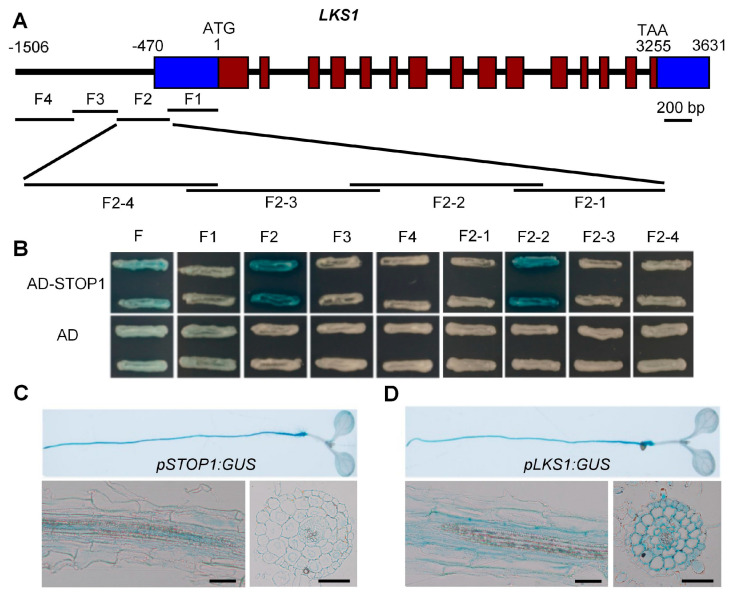
STOP1 can bind to the *LKS1* promoter region in vitro. (**A**) Schematic structure of *LKS1* gene and the fragments segmented from the *LKS1* promoter. The adenine residue of the translational start codon (ATG) is assigned position +1. The blue boxes represent the 5′-UTR (left, −470 to −1) and 3′-UTR (right, 3256 to 3631), respectively. The promoter region F was divided into four fragments F1 (−382 to −1 bp), F2 (−757 to −364 bp), F3 (−1078 to −738 bp), and F4 (−1506 to −1061 bp). Then, F2 was divided into F2-1 (−460 to −361 bp), F2-2 (−560 to −441 bp), F2-3 (−660 to −541 bp), and F2-4 (−760 to −641 bp). (**B**) Yeast-one-hybrid assays showing that STOP1 can bind to the fragments of *LKS1* promoter. The coding sequence of *STOP1* was constructed into the vector pB42AD. AD refers to the empty vector expressing the AD domain alone. *LacZ* was used as a reporter gene, driven by the fragments of *LKS1* promoter in yeast. (**C,D**) GUS staining analyses of *STOP1* (**C**) and *LKS1* (**D**) expression in 7 day old *Arabidopsis* seedlings. The lower panels show the vertical (left) and cross-sections (right) of roots, respectively. Bars = 50 μm.

Our previous study showed that the *LKS1* transcript is strongly increased under low-K^+^ (LK) conditions [11], suggesting that there may exist some transcription factors that can positively regulate *LKS1* transcription in *Arabidopsis* response to LK stress. In order to identify these transcription factors, a high-throughput screening was performed using the yeast-one-hybrid (Y1H) method [36]. First, the full-length (1506 bp) promoter region of the *LKS1* gene was cloned and divided into four overlapping fragments (F1 to F4) (Figure 1A). Then, these four fragments were used as baits to screen the transcription factor library containing 1589 *Arabidopsis* transcription factors from 62 transcription factor families [36]. Here, *HIS3* was used as a reporter gene. Finally, a zinc finger protein named STOP1 was isolated [25]. As shown in Figure S1, STOP1 could bind to the F2 region (−757 to −364 bp) of the *LKS1* promoter. To confirm the binding activity, the F2 region was divided into four small fragments (F2-1 to F2-4) that were further tested in a Y1H assay (Figure 1A). Here, *LacZ* was used as a reporter gene. We found that STOP1 could only bind to the F2 and F2-2 fragments (−560 to −441 bp) (Figure 1B). The screening results revealed that STOP1 may be the transcription factor that regulates *LKS1* expression.

If STOP1 regulates *LKS1* transcription, these two genes should have similar tissue expression patterns. Thus, their expression patterns were determined in *Arabidopsis* using the transgenic GUS lines (*pSTOP1:GUS* and *pLKS1:GUS*) driven by the *STOP1* or *LKS1* promoter. GUS staining showed that both genes were primarily expressed in *Arabidopsis* root at the seedling stage (Figure 1C,D). They were broadly expressed in root hair, epidermis, cortex, endodermis, and stele (Figure 1C,D). This similar expression pattern suggested that these two genes may work together in *Arabidopsis* root.

### 2.2. stop1 Mutants Are Sensitive to LK Stress

**Figure 2:**
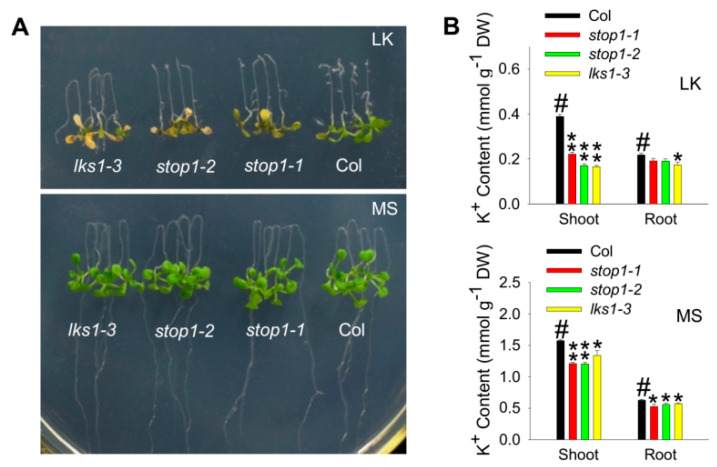
*stop1* mutants are sensitive to low-K^+^ stress. (**A**) Phenotype test of *stop1* and *lks1* mutants on MS (20 mM K^+^) and LK (0.1 mM K^+^) medium. Seeds were germinated on MS medium for 5 days, and then the seedlings were transferred to MS or LK medium for 10 days. (**B**) K^+^ content measurement of indicated plants shown in (**A**) after being transferred to MS or LK medium for 7 days. Data are shown as means ± SE (*n* = 4 as described in Section 4). Student’s *t*-test (* *p* < 0.05 and ** *p* < 0.01) was used to analyze statistical significance; # represents the control.

In a previous study, we found that *LKS1* is an essential component in the LK signaling pathway, and *lks1* mutants showed a leaf chlorosis phenotype when grown on LK (0.1 mM K^+^) medium [11]. To verify if *STOP1* is also involved in this LK signal pathway by regulating *LKS1* gene, two independent *stop1* mutants were obtained, and their LK phenotypes were tested in this study. The *stop1-1* mutant contains a C796T substitution that causes a H266Y change in amino-acid sequence located at the C2H2-type zinc finger DNA-binding domain (Figure S2A,B). On the other hand, *stop1-2* is a T-DNA insertion mutant (Figure S2A,C,D). These two alleles were verified as loss-of-function mutants [25]. Phenotype test showed that both *stop1* mutants exhibited a leaf chlorosis phenotype when grown on LK (0.1 mM K^+^) medium for 10 days, which was similar to the *lks1-3* mutant (Figure 2A). When grown on MS medium (high K^+^ condition, 20 mM), all tested plants did not show any obvious phenotypic difference (Figure 2A). K^+^ content measurement indicated that, under LK conditions, the *stop1* mutants and *lks1-3* mutant had much lower K^+^ contents in the shoot than wild type (Col) (Figure 2B), which was consistent with the shoot sensitive phenotype of these mutants on LK medium (Figure 2A).

We also constructed the complementation lines (*stop1-2*/*pSTOP1:STOP1*) using the *STOP1* genomic sequence. Two independent transgenic lines (*COM1* and *COM2*) can fully complement the sensitive phenotype of the *stop1* mutant on LK medium (Figure S3A). In addition, the K^+^ contents in these two complementation lines were restored to the wild-type level (Figure S3B). These results indicated that loss of function of *STOP1* leads to a reduction in plant K^+^ content and results in the LK sensitive phenotype.

### 2.3. STOP1 Positively Regulates LKS1 Transcription

**Figure 3:**
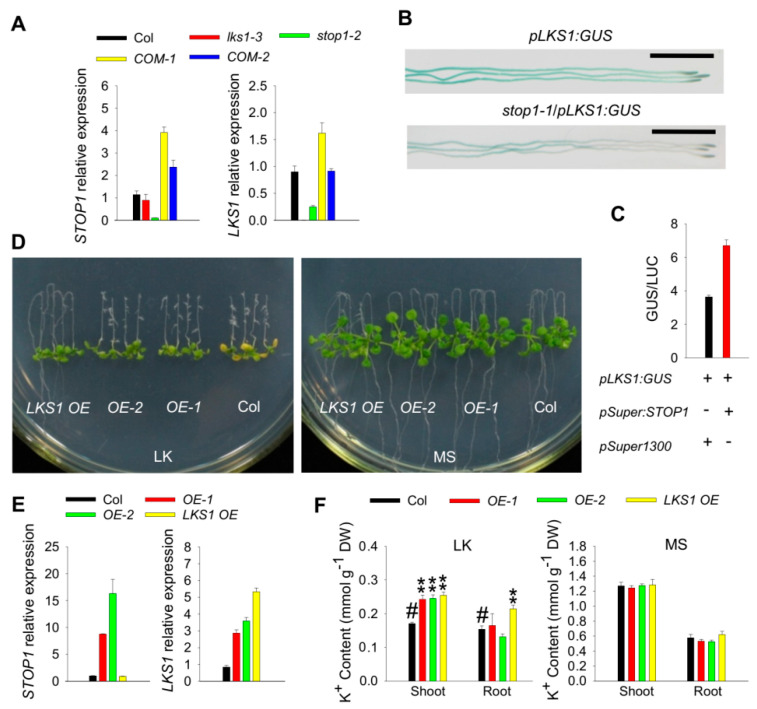
STOP1 positively regulates *LKS1* transcription. (**A**) RT-qPCR analyses of *STOP1* (left) and *LKS1* (right) expression in wild type (Col), *lks1-3*, *stop1-2*, and complementation lines *stop1-2*/*pSTOP1:STOP1* (*COM-1* and *COM-2*). Seedlings were germinated on MS medium for 7 days, and then roots were collected and used for RT-qPCR assays. Data are shown as means ± SE (*n* = 3). (**B**) GUS staining showing *LKS1* expression in the roots of wild type and *stop1-1* mutant. Seedlings were germinated on MS medium for 6 days, and then used for GUS staining. Scale bars = 2 mm. (**C**) GUS activity measurement in tobacco leaves after transient expression of *pLKS1:GUS* and *pSuper:STOP1*. *LUC* was used as an internal control. Data are means ± SE (*n* = 4). (**D**) Phenotype comparison among *LKS1* overexpressing line (*LKS1 OE*), *STOP1* overexpressing lines (*OE-1* and *OE-2*), and wild-type (Col) plants. The 5 day old seedlings were transferred to MS or LK medium for 12 days. (**E**) RT-qPCR analyses of *STOP1* (left) and *LKS1* (right) expression in the roots of tested materials as indicated. Seedlings were germinated on MS medium for 7 days, and then roots were collected and used for RT-qPCR assays. Data are presented as means ± SE (*n* = 3). (**F**) K^+^ content measurement of indicated plants shown in (**D**). K^+^ contents were measured after seedlings were transferred to MS or LK medium for 10 days. Data are shown as means ± SE (*n* = 4). Student’s *t*-test (** *p* < 0.01) was used to analyze statistical significance; # represents the control.

The *stop1* and *lks1* mutants showed a similar leaf chlorosis phenotype on LK medium, suggesting that *STOP1* may be a positive regulator of the *LKS1* gene. Then, the expression levels of both *STOP1* and *LKS1* were determined in *Arabidopsis* root. RT-qPCR results showed that the transcriptional level of *LKS1* was significantly reduced in the *stop1-2* mutant (Figure 3A). When *STOP1* transcripts were restored in the complementation lines (*COM1* and *COM2*), *LKS1* transcripts were subsequently resumed (Figure 3A). In addition, we also crossed the *pLKS1:GUS* line with the *stop1-1* mutant to test *LKS1* expression. GUS staining indicated that *LKS1* expression level was greatly reduced in *stop1-1* mutant root (Figure 3B). To further confirm this positive regulation, we performed transient expression experiments in tobacco (*Nicotiana benthamiana*) leaves. When *pSuper:STOP1* and *pLKS1:GUS* were co-expressed in tobacco leaves, the GUS activity was significantly increased (Figure 3C).

In order to investigate the physiological role of this positive regulation in plants, *STOP1* overexpressing lines were constructed, and two independent transgenic lines (*STOP1 OE1* and *OE2*) were obtained. When grown on LK medium for 12 days, the wild-type shoot became yellow, while the shoots of *STOP1 OE* lines remained green (Figure 3D). This phenotype was very similar to the shoot tolerant phenotype of the *LKS1 OE* line (Figure 3D), and the *LKS1* transcripts were indeed significantly increased in the *STOP1 OE* lines (Figure 3E). In addition, K^+^ content measurement further confirmed that the shoot tolerant phenotypes of *STOP1* and *LKS1* overexpressing plants were due to the increased shoot K^+^ contents under LK conditions (Figure 3F). All these data clearly demonstrated that STOP1 positively regulates *LKS1* transcription in *Arabidopsis* root and subsequently promotes LKS1-mediated K^+^ uptake.

### 2.4. STOP1 Acts Upstream of LKS1

**Figure 4:**
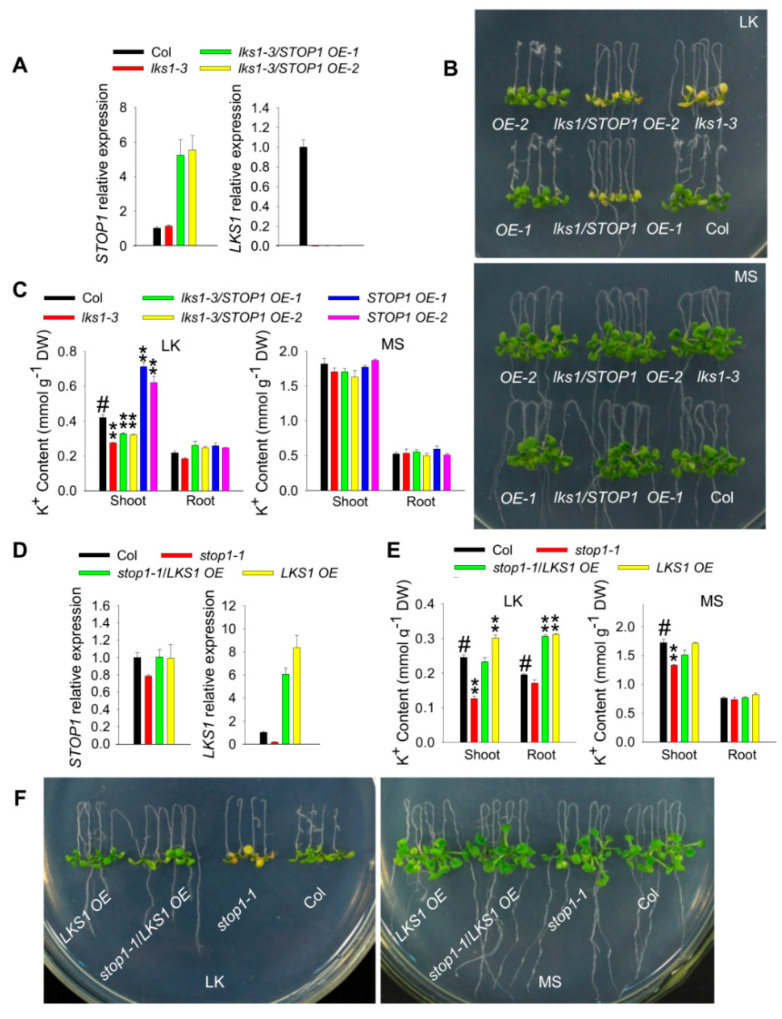
*STOP1* acts upstream of *LKS1.* (**A**) RT-qPCR analyses of *STOP1* (left) and *LKS1* (right) expression in the roots of various plants. Data are presented as means ± SE (*n* = 3). (**B**) Phenotype comparison of wild type (Col), *lks1-3*, *STOP1 OE* (*OE-1* and *OE-2*), and *lks1-3*/*STOP1 OE* (*lks1*/*STOP1 OE-1* and *lks1*/*STOP1 OE-2*). The 5 day old seedlings were transferred to MS or LK medium for 10 d. (**C**) K^+^ content measurement of indicated plants shown in (**B**) after being transferred to MS or LK medium for 7 days. Data are shown as means ± SE (*n* = 3). Student’s *t*-test (** *p* < 0.01) was used to analyze statistical significance; # represents the control. (**D**) RT-qPCR analyses of *STOP1* (left) and *LKS1* (right) expression in the roots of tested materials as indicated. Data are presented as means ± SE (*n* = 3). (**E**) K^+^ content measurement of indicated plants shown in (**F**) after being transferred to MS or LK medium for 7 days. Data are shown as means ± SE (*n* = 4). Student’s *t* test (** *p* < 0.01) was used to analyze statistical significance; # represents the control. (**F**) Phenotype comparison among *stop1-1/LKS1 OE*, *stop1-1*, *LKS1 OE*, and wild-type plants. The 5 day old seedlings were transferred to MS or LK medium for 10 days.

To investigate the relationship between *STOP1* and *LKS1*, a genetic approach was used. We constructed *STOP1 OE* lines in the *lks1-3* mutant background (Figure 4A) and tested their phenotypes. As we know, the *STOP1 OE* lines (in wild-type background) showed an LK tolerant phenotype (Figure 3D). However, the two independent transgenic lines *lks1-3*/*STOP1 OE-1* and *lks1-3*/*STOP1 OE-2* displayed an identical leaf chlorosis phenotype to the *lks1-3* mutant under LK conditions (Figure 4B). Consistent with the phenotype, the shoot K^+^ contents in these two transgenic lines were close to the *lks1-3* mutant (Figure 4C).

Conversely, *stop1-1*/*LKS1 OE* plants were also constructed by crossing the *stop1-1* mutant with the *LKS1 OE* plants (Figure 4D). Phenotype test showed that this crossing line fully complemented the leaf chlorosis phenotype of *stop1-1* mutant under LK conditions (Figure 4F). The K^+^ content in this crossing line was significantly increased under LK conditions (Figure 4E). The *lks1 stop1-2* double mutants were also constructed by knocking *LKS1* out in the *stop1-2* mutant using the Crispr/Cas9 technique. The double mutant plants showed a similar phenotype to the *lks1-3* mutant (Figure S5). This genetic evidence confirmed that *STOP1* and *LKS1* function in the same LK signaling pathway, and STOP1 should be the upstream transcription factor of *LKS1* gene.

### 2.5. STOP1 Directly Binds to the LKS1 Promoter

**Figure 5:**
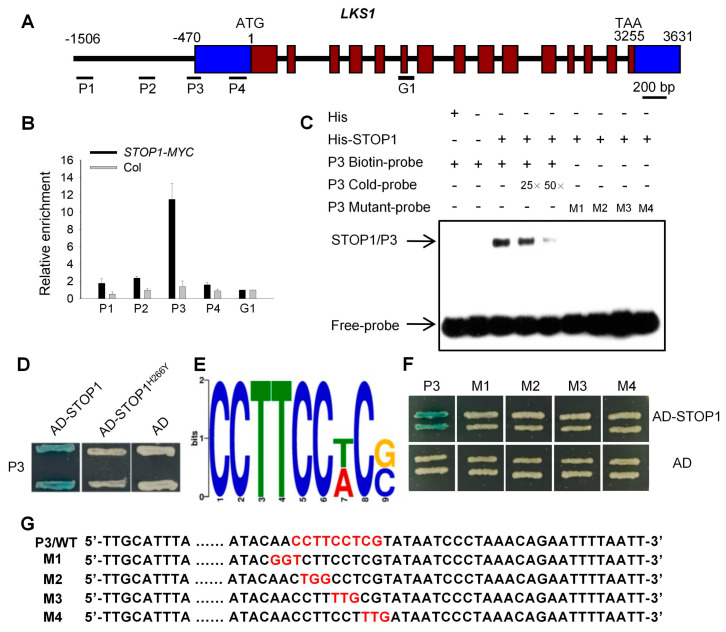
STOP1 binds to the *LKS1* promoter in vivo. (**A**) Diagram of the *LKS1* promoter and genomic region. Four fragments within the *LKS1* promoter, P1 (−1506 to −1366 bp), P2 (−974 to −837 bp), P3 (−560 to −441 bp), and P4 (−200 to −51 bp), were used in ChIP-qPCR assay. The fragment G1 (1223 to 1357 bp) was used as a negative control. (**B**) ChIP-qPCR analyses of STOP1 binding activity to the *LKS1* promoter. The roots of 12 day old seedlings (*pSuper:STOP1-MYC* and Col) were harvested for ChIP assay using anti-MYC antibody. Then, the precipitated DNA was analyzed by qPCR assay. Data are means ± SE (*n* = 3). (**C**) EMSA analyses showing the DNA-binding activity of STOP1 to the P3 fragment within *LKS1* promoter. The purified His-STOP1 proteins were incubated with P3 probes labeled with biotin. Excessive unlabeled P3 probes (P3 Cold-probe) were added to compete with biotin-labeled probes. The mutant probes of P3 (M1 to M4) were shown in (**G**). (**D**) Yeast-one-hybrid analyses of STOP1^H266Y^ binding activity to the P3 fragment. STOP1^H266Y^ is the point mutation of STOP1 in the *stop1-1* mutant. (**E**) The core binding sequence in the P3 fragment. The MEME online software was used to predict core binding sequence. (**F**) Yeast-one-hybrid assays showing that STOP1 could not bind to the mutated fragments of P3 (M1 to M4). (**G**) Four mutated fragments of P3 (M1 to M4) in the binding sequence. The core binding sequence was shown in red.

To determine whether STOP1 can directly bind to the *LKS1* promoter in planta, a chromatin immunoprecipitation (ChIP) assay was performed using the *pSuper:STOP1-MYC* transgenic plants. We designated four fragments (P1 to P4) within the *LKS1* promoter, and one fragment (G1) within *LKS1* exon and intron regions was used as the negative control (Figure 5A). The ChIP-qPCR results indicated that STOP1 could only bind to the P3 fragment (Figure 5B). This P3 (−560 bp to −441 bp) is the same as the F2-2 fragment used in the Y1H assay (Figure 1B). In addition, the DNA-binding activity of STOP1 was also confirmed using the electrophoresis mobility shift assay (EMSA). As shown in Figure 5C, STOP1 bound to the P3 probe labeled with biotin. The binding signal was gradually reduced by addition of unlabeled competitive probes. We also found that the mutated protein STOP1^H266Y^ from the *stop1-1* mutant could not bind to the P3 fragment in the Y1H assay (Figure 5D), indicating that STOP1^H266Y^ loses DNA-binding activity (Figure S6). These data demonstrated that STOP1 directly binds to the *LKS1* promoter in vivo.

Previous data suggested that the binding site of STOP1 should be within the F2 fragment (Figure 1A,B). Therefore, we used MEME online software (http://meme-suite.org/tools/meme (3 January 2021)) to predict the core binding sequence within this fragment, and the sequence CCTTCC[T/A]C[G/C] had the highest score (Figure 5E). According to this prediction, we supposed that the sequence CCTTCCTCG within the P3 fragment was the binding site of STOP1. To verify this core sequence, Y1H and EMSA assays were performed, and four mutated fragments (M1 to M4) of P3 were designed (Figure 5G). The results from both Y1H and EMSA assays indicated that STOP1 no longer bound to the P3 fragment when the core sequence was mutated (Figure 5C,F). These data confirmed that CCTTCCTCG is the core *cis*-element on the *LKS1* promoter that is the binding site of the STOP1 protein.

### 2.6. K^+^ Uptake Is Impaired in Stop1 Mutant

**Figure 6:**
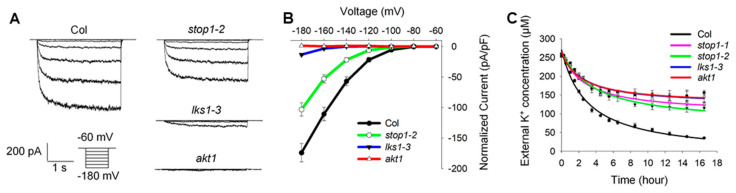
K^+^ uptake is impaired in *stop1* mutant. (**A**) Patch-clamp whole-cell recordings of the inward K^+^ currents in *Arabidopsis* root-cell protoplasts. The plant materials used for root-cell protoplasts isolation are indicated. The voltage protocols, as well as time and current scale bars, for the recordings are shown. (**B**) I–V relationship of the steady-state whole-cell currents in root cells. The data are derived from the recordings as shown in (**A**) and are presented as means ± SE (Col, *n* = 61; *stop1-2*, *n* = 51; *lks1-3*, *n* = 51; *akt1*, *n* = 24). (**C**) Comparison of K^+^ uptake via K^+^ depletion method among Col, *lks1*, *akt1*, and *stop1* mutants. Data are shown as means ± SE (*n* = 3).

Since LKS1 positively regulates AKT1- and HAK5-mediated K^+^ uptake, we determined AKT1 channel activity and K^+^ uptake capacity in *stop1* mutant in vivo. Patch-clamp whole-cell recordings were conducted to test AKT1 channel activity in *Arabidopsis* root cell protoplasts. As shown in Figure 6A,B, the inward K^+^ currents in *akt1* mutant almost disappeared as in previous reports [11]. Similarly, *lks1-3* mutant only showed very weak K^+^ currents. Compared with wild type (−174.02 ± 14.98 pA/pF at −180 mV), the AKT1-mediated inward K^+^ currents were significantly reduced in *stop1-2* mutant (−103.21 ± 10.76 pA/pF at −180 mV), but not absent, because the leak expression of *LKS1* in *stop1* mutant may also partially activate AKT1. The statistical data further confirmed that the inward K^+^ current density (at −180 mV) in the *stop1* mutant was significantly reduced (Table 1). In addition, we also determined the K^+^ uptake capacities of these mutants using the K^+^-depletion method. As shown in Figure 6C, all tested mutants showed reduced K^+^ uptake rates and capacities compared with wild type. All these results demonstrated that disruption of *STOP1* affects root K^+^ uptake by reducing *LKS1* expression.

### 2.7. The Expression of Both STOP1 and LKS1 Is Induced after LK Stress

**Figure 7:**
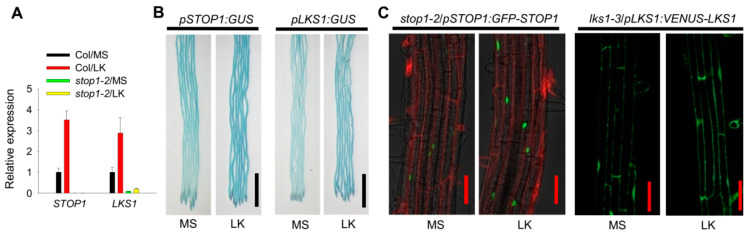
The expression of both *STOP1* and *LKS1* is induced after low-K^+^ stress. (**A**) RT-qPCR analyses of *STOP1* and *LKS1* expression in wild type (Col) and *stop1-2* mutant after LK stress. The 5 day old seedlings were transferred to MS or LK medium for 1 day. Then, the roots were collected and used for RT-qPCR assays. Data are means ± SE (*n* = 4). (**B**) GUS staining analyses of *STOP1* and *LKS1* expression after LK treatment. The 5 day old seedlings were transferred to MS or LK medium for 1 day, and then used for GUS staining. Scale bars = 2 mm. (**C**) Fluorescence observation showing the protein expression of GFP-STOP1 and VENUS-LKS1 after LK stress. The red fluorescence was due to the propidium iodine staining. The 5 day old seedlings were transferred to MS or LK medium for 1 day, and then used for fluorescence observation. Scale bars = 50 μm.

Our previous study showed that *LKS1* expression can be strongly induced after LK stress [11]. Since STOP1 positively regulates *LKS1* transcription, how does *STOP1* respond to LK stress? Here, we determined the transcriptional levels of *STOP1* and *LKS1* genes in *Arabidopsis* response to LK stress. RT-qPCR results showed that the transcripts of both *STOP1* and *LKS1* genes were significantly increased in wild-type root, when plants were transferred from MS medium to LK medium for 1 day (Figure 7A). In the *stop1-2* mutant, the *LKS1* transcript was no longer induced (Figure 7A), suggesting that the induction of *LKS1* transcription requires *STOP1*. These results indicated that *STOP1* can also be regulated at transcriptional level.

Furthermore, we also used the *pSTOP1:GUS* and *pLKS1:GUS* plants to determine the expression levels of *STOP1* and *LKS1* genes after LK stress. GUS staining results indicated that the transcription of both *STOP1* and *LKS1* was upregulated in *Arabidopsis* root after LK stress (Figure 7B). The LK-induced pLKS1:GUS activity was significantly impaired in the *stop1-1/pLKS1:GUS* line (Figure S7A). In addition, the transgenic plants *stop1-2*/*pSTOP1:GFP-STOP1*, *stop1-2*/*pUBQ:GFP-STOP1* [28], and *lks1-3*/*pLKS1:VENUS-LKS1* were used to indicate the protein levels of STOP1 and LKS1 in intact plant roots. Fluorescence observation clearly showed that, after LK stress, GFP-STOP1 proteins were accumulated in nucleus, while VENUS-LKS1 protein level was also increased in cytoplasm (Figure 7C and Figure S7B–D). It is suggested that the increased LKS1 expression is due to the increased STOP1 expression. All these data demonstrated that LK stress promotes *STOP1* and *LKS1* expression at both transcriptional and protein levels. This STOP1/LKS1 regulation pathway positively responds to LK stress.

### 2.8. STOP1 and LKS1 Are Involved in Both Low-K^+^ and High-NH_4_^+^ Responses

**Figure 8:**
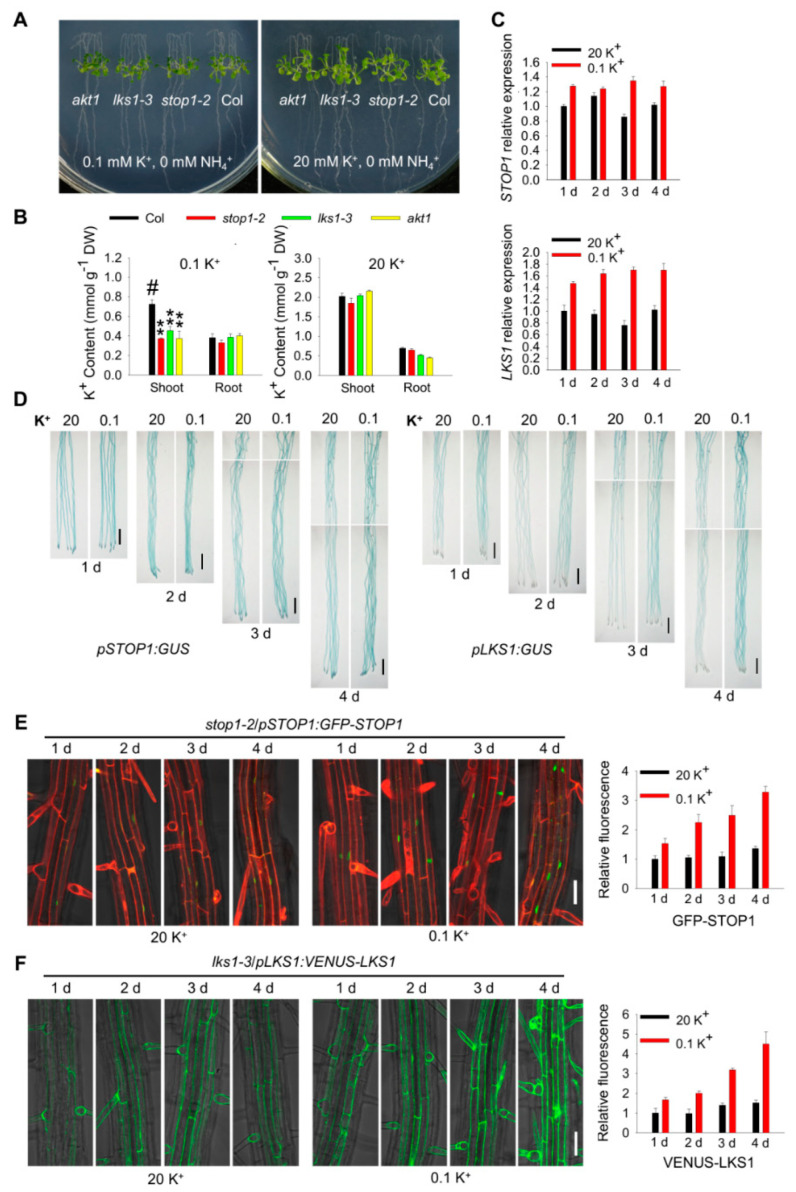
*STOP1* and *LKS1* are involved in low-K^+^ response in the absence of NH_4_^+^. (**A**) Phenotype test of *akt1*, *stop1*, and *lks1* mutants on high-K^+^ (20 mM) and low-K^+^ (0.1 mM) medium in the absence of NH_4_^+^. Seeds were germinated on high-K^+^ (20 mM) medium for 5 days, and then the seedlings were transferred to high- or low-K^+^ medium for 10 days. (**B**) K^+^ content measurement of indicated plants shown in (**A**) after being transferred to high- or low-K^+^ medium for 7 days. Data are shown as means ± SE (*n* = 4). Student’s *t*-test (** *p* < 0.01) was used to analyze statistical significance; # represents the control. (**C**) RT-qPCR analyses of *STOP1* and *LKS1* expression in low-K^+^ response in the absence of NH_4_^+^. The 5 day old seedlings were transferred to high- or low-K^+^ medium for the indicated times. Then, roots were collected and used for RT-qPCR assays. Data are shown as means ± SE (*n* = 4). (**D**) GUS staining showing *STOP1* and *LKS1* expression in low-K^+^ response in the absence of NH_4_^+^. The 5 day old seedlings were transferred to high- or low-K^+^ medium for the indicated times, and then used for GUS staining. Scale bars = 2 mm. (**E**,**F**) Fluorescence observation showing the protein expression of GFP-STOP1 and VENUS-LKS1 after low-K^+^ stress in the absence of NH_4_^+^. The relative fluorescence intensity of GFP-STOP1 (*n* = 10) and VENUS-LKS1 (*n* = 3) was calculated. The red fluorescence was due to the propidium iodine staining. The 5 day old seedlings were transferred to high- or low-K^+^ medium for the indicated times, and then used for fluorescence observation. Scale bars = 50 μm.

Following our previous LK assays, KNO_3_ and KH_2_PO_4_ in MS medium were replaced by NH_4_NO_3_ and NH_4_H_2_PO_4_ to reduce K^+^ concentration [11]. Therefore, high NH_4_^+^ (30 mM) was also introduced into the LK medium. Our previous study showed that the low-K^+^ sensitive phenotype (leaf chlorosis) of *lks1* mutants is dependent on both low K^+^ and high NH_4_^+^ [11], and it is also true for *stop1* mutant (Figure S9A). Since NH_4_^+^ and K^+^ can compete with one another for uptake, it is necessary to clarify whether *STOP1* and *LKS1* are involved in the low-K^+^ response in the absence of NH_4_^+^. Therefore, NH_4_^+^ in the LK medium was totally removed. Under these conditions (0.1 mM K^+^, 0 mM NH_4_^+^), all the mutants (*akt1*, *lks1*, and *stop1*) did not show obvious phenotypes compared with wild type (Figure 8A). However, the shoot K^+^ content of all these mutants was significantly reduced (Figure 8B). In addition, the K^+^ uptake capacities of these mutants were all impaired when tested in the patch-clamp recording and K^+^-depletion assay (Figure 6), and no NH_4_^+^ was present in these assays. These data suggested that STOP1 indeed regulates LKS1- and AKT1-mediated K^+^ uptake in the absence of NH_4_^+^. Both RT-qPCR and GUS staining results indicated that this low-K^+^ condition (0.1 mM K^+^, 0 mM NH_4_^+^) could induce the upregulation of both *STOP1* and *LKS1* transcripts, although the induction needed more time (Figure 8C,D). Consistent with the transcriptional levels, after this LK treatment, STOP1 and LKS1 proteins accumulated in the nucleus and cytoplasm, respectively (Figure 8E,F). These data demonstrated that STOP1 and LKS1 are indeed involved in the low-K^+^ response in the absence of NH_4_^+^.

**Figure 9:**
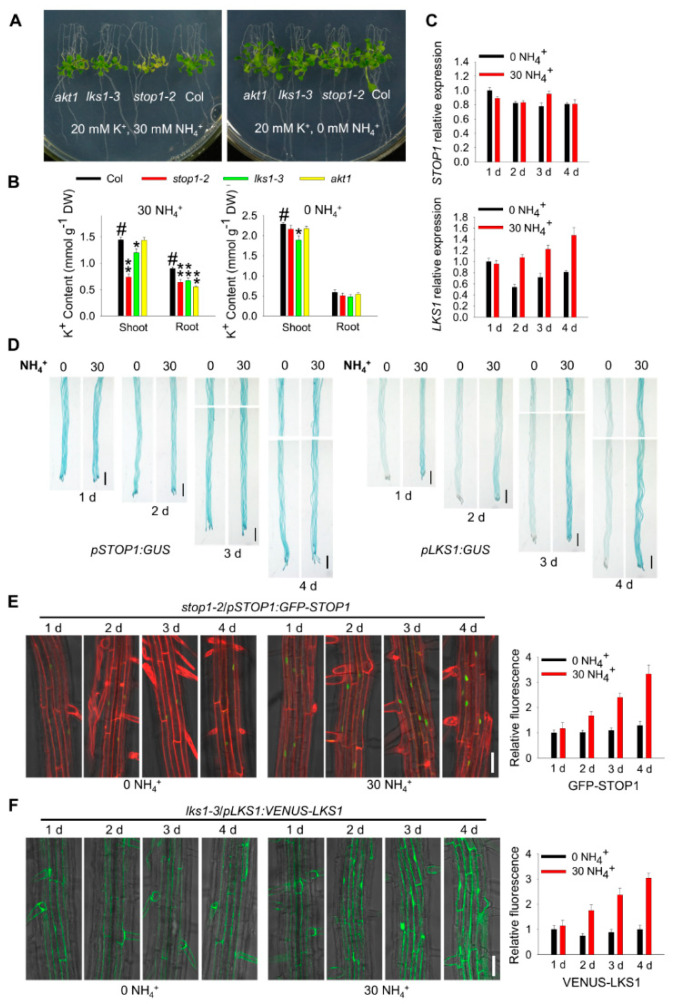
STOP1 and LKS1 are involved in the high-NH_4_^+^ response. (**A**) Phenotype test of *akt1*, *stop1*, and *lks1* mutants on high-NH_4_^+^ (30 mM) and low-NH_4_^+^ (0 mM) medium. Seeds were germinated on low-NH_4_^+^ (0 mM) medium for 5 days, and then the seedlings were transferred to high o-r low-NH_4_^+^ medium for 10 days. (**B**) K^+^ content measurement of indicated plants shown in (**A**) after being transferred to high- or low-NH_4_^+^ medium for 7 days. Data are shown as means ± SE (*n* = 4). Student’s *t*-test (* *p* < 0.05 and ** *p* < 0.01) was used to analyze statistical significance; # represents the control. (**C**) RT-qPCR analyses of *STOP1* and *LKS1* expression in high-NH_4_^+^ response. The 5 day old seedlings were transferred to high- or low-NH_4_^+^ medium for the indicated times. Then, roots were collected and used for RT-qPCR assays. Data are shown as means ± SE (*n* = 4). (**D**) GUS staining showing *STOP1* and *LKS1* expression in the high-NH_4_^+^ response. The 5 day old seedlings were transferred to high- or low-NH_4_^+^ medium for the indicated times, and then used for GUS staining. Scale bars = 2 mm. (**E**,**F**) Fluorescence observation showing the protein expression of GFP-STOP1 and VENUS-LKS1 after high-NH_4_^+^ stress. The relative fluorescence intensity of GFP-STOP1 (*n* = 10) and VENUS-LKS1 (*n* = 5) was calculated. The red fluorescence was due to the propidium iodine staining. The 5 day old seedlings were transferred to high- or low-NH_4_^+^ medium for the indicated times, and then used for fluorescence observation. Scale bars = 50 μm.

We also analyzed the roles of *STOP1* and *LKS1* in the high-NH_4_^+^ response. Under high-NH_4_^+^ conditions (5/10/20 mM K^+^, 30 mM NH_4_^+^), *akt1* and *lks1* mutants did not show obvious different phenotypes compared with wild type. However, the *stop1* mutant displayed a light-yellow shoot phenotype (Figure 9A and Figure S9B,C), suggesting that STOP1 is involved in the high-NH_4_^+^ response. The K^+^ content of all these mutants was significantly reduced under high-NH_4_^+^ conditions, and the shoot K^+^ content of *stop1* mutant was much lower than that of *lks1* (Figure 9B). It is suggested that the yellow shoot phenotype of *stop1* could be due to the NH_4_^+^-induced shoot K^+^ content reduction. According to the phenotypes and K^+^ content, STOP1 may regulate some other K^+^/NH_4_^+^-related genes in addition to *LKS1*. RT-qPCR and GUS staining results indicated that the transcripts of *STOP1* were not affected after high-NH_4_^+^ treatment, while the *LKS1* transcript level was significantly upregulated (Figure 8C,D). Both STOP1 and LKS1 proteins were accumulated after high-NH_4_^+^ treatment (Figure 8E,F).

All these data demonstrated that *STOP1* and *LKS1* are involved in both low-K^+^ and high-NH_4_^+^ responses. The transcriptional and translational levels of *LKS1* are upregulated by low-K^+^ and high-NH_4_^+^. The transcript level of *STOP1* is only upregulated by low K^+^ rather than high NH_4_^+^; however, the accumulation of STOP1 protein is required for upregulation of the *LKS1* transcript in low-K^+^ and high-NH_4_^+^ responses.

## 3. Discussion

The protein kinase LKS1/CIPK23 has been reported to play essential roles in diverse signaling pathways. Under LK conditions, LKS1 phosphorylates the K^+^ channel AKT1 [11], as well as the K^+^ transporter HAK5 [21], to enhance K^+^ uptake in *Arabidopsis* root cells. Therefore, the plant tolerance to LK stress is strengthened. In addition, LKS1/CIPK23 also regulates the activity of nitrate transporter NRT1.1/CHL1. NRT1.1 is involved in both high- and low-affinity NO_3_^−^ uptake [37,38]. Under low NO_3_^−^ concentrations, CIPK23 phosphorylates NRT1.1, via which NRT1.1 is converted into a high-affinity nitrate transporter to adapt the reduced NO_3_^−^ level [23]. A recent report showed that CIPK23 also inhibits the activity of ammonium transporter AMT1;1/1;2 to avoid NH_4_^+^ toxic accumulation under high-NH_4_^+^ conditions [24]. Therefore, LKS1/CIPK23 is a key component to coordinate potassium and nitrogen balance in plant roots. More importantly, the transcription of *LKS1*/*CIPK23* can respond to external K^+^, NO_3_^−^, and NH_4_^+^ concentrations [11,23,24], suggesting that the transcriptional regulation of *LKS1*/*CIPK23* is essential in plant response to external potassium and nitrogen levels. The present study not only reveals the transcriptional regulation in plant LK response, but also may provide some clues to understand nitrogen response.

Previous studies have demonstrated that STOP1 is essential for proton and Al^3+^ tolerance by regulating the expression of malate transporter *ALMT1* (*ALUMINUM-ACTIVATED MALATE TRANSPORTER* *1*) and citrate transporter *MATE* (*MULTIDRUG AND TOXIC COMPOUND EXTRUSION*) [25,26,39]. In addition, STOP1 also regulates root cell elongation under low Pi stress [28]. In the present study, we demonstrate that STOP1 is involved in the low-K^+^ response by regulating *LKS1/CIPK23* transcription. A recent report also found that STOP1 regulates salt and drought tolerance by modulating *LKS1/CIPK23* transcription [40]. All this evidence supports a conclusion that STOP1 functions as a key node that controls ion homeostasis when plants are subjected to nutrient or ion stresses. The STOP1/LKS1 regulatory pathway may play more important roles in the plant nutrient/ion regulatory network.

STOP1 is an important component to regulate the transcription of downstream target genes; however, how is the *STOP1* gene or protein regulated? Under low pH or Al^3+^ stresses, the transcriptional level of *STOP1* is not affected [25]. In addition, the *STOP1* transcript is not changed under low-phosphorus conditions [28,29]. A recent study showed that the F-box protein RAE1 (Regulation of Atalmt1 Expression 1) directly interacts with STOP1 and controls the stability of STOP1 proteins through the 26S proteasome pathway in Al^3+^ resistance [41]. These studies suggest that STOP1 is regulated at the post-translational level rather than the transcriptional level. Here, we found that STOP1 protein is accumulated after low-K^+^ or high-NH_4_^+^ stresses, and the *RAE1 OE* plants showed similar phenotypes to *stop1* mutant under our LK conditions (Figure 8, Figure 9 and Figure S8E). It is noteworthy that the transcript level of *STOP1* was upregulated by low K^+^ but not high NH_4_^+^ (Figure 8 and Figure 9). Therefore, *STOP1* can be regulated at both transcriptional and post-translational levels. We suggest that the different regulatory mechanisms may depend on the different upstream stress signals. STOP1 is involved in Al^3+^ resistance by upregulating *ALMT1* transcription. In the present study, we found that STOP1 participates in the LK response by elevating *LKS1* transcription. However, the *almt1* mutant did not show any sensitive or tolerant phenotype when grown on LK medium (Figure S8C), suggesting that STOP1/ALMT1 and STOP1/LKS1 are two independent signaling pathways involved in different stress responses. In addition, 5 mM MES was added to LK medium to stabilize pH, and it could be seen that *stop1*, *lks1* and *akt1* still showed LK sensitive phenotypes (Figure S8F). The results showed that the sensitive phenotype of the *stop1* mutant was not mainly caused by the decreased pH in the medium.

A previous study showed that STOP1 regulates *ALMT1* transcription by binding to the sequence GGGGAGGGC in the *ALMT1* promoter [42]. However, we found that the binding site of STOP1 on the *LKS1* promoter is the sequence CCTTCCTCG (Figure 5). A recent study indicated that STOP1 can also bind to the *RAE1* promoter at the sequence CCTTCCTCG [41], suggesting that STOP1 binds to the same *cis*-element in the *LKS1* and *RAE1* promoters. In addition, this binding site was also confirmed by a recent report [40]. Obviously, STOP1 regulates different target genes by binding to different *cis*-elements in promoter regions. However, the mechanisms underlying how STOP1 responds to different stress signals and recognizes different *cis*-elements still need to be further investigated.

According to the expression analyses, the transcripts of *LKS1* were extremely low in *stop1* mutants compared with wild type (Figure 3A, Figure 4D and Figure 7A), suggesting that STOP1 should be the major transcription factor regulating *LKS1*. In addition, the STOP1 homolog, STOP2, is not involved in the LK stress response, because the *stop2* mutant did not show any different phenotype compared with the wild type when grown on LK medium (Figure S8D).

Low K^+^ or high NH_4_^+^ alone did not cause the obvious leaf chlorosis phenotype in *lks1* and *akt1* mutants in our transfer assays, although the K^+^ content in mutant was significantly reduced (Figure 8 and Figure 9). The presence of NH_4_^+^ could inhibit K^+^ uptake, subsequently enhancing the leaf chlorosis phenotype of *akt1* and *lks1* mutants under low-K^+^ conditions (Figure S9A; [13]). Therefore, NH_4_^+^ used in the LK medium makes the phenotype more visible; however, it also causes NH_4_^+^ toxicity. A recent study revealed that CIPK23 inhibits NH_4_^+^ transport under NH_4_^+^ toxic conditions [24]. Here, our data also demonstrate that STOP1/LKS1 is involved in the NH_4_^+^ response.

NH_4_^+^ can inhibit primary root growth of plants under low-K^+^ conditions [9,13]. Along with the increment in NH_4_^+^, the primary root growth of wild-type and mutant plants (*stop1*, *lks1*, and *akt1*) was gradually inhibited (Figure S9A). Wild-type root growth was restricted in low-K^+^ and high-NH_4_^+^ conditions; however, *lks1* and *akt1* root could still grow (Figure 2B) [11]. Our previous study demonstrated that AKT1 is involved in low-K^+^ sensing (presence of NH_4_^+^) in *Arabidopsis* root and subsequently regulates root growth by modulating PIN1 (PIN-FORMED 1) degradation and auxin redistribution in root [43]. The *akt1* mutant root cannot respond to external LK stress; *lks1* and *cbl1 cbl9* mutants display a similar root phenotype in this regard. Therefore, loss of function of *AKT1*, *LKS1*, and *CBL1/9* leads to the root growth phenotype under this LK conditions. We noticed that the primary root of *stop1* mutants could not grow under LK conditions, which is similar to wild type. In *stop1* mutant, *LKS1* expression was significantly reduced (Figure 3A), which impaired AKT1- and HAK5-mediated K^+^ uptake (Figure 6C, [21]), resulting in a leaf chlorosis phenotype (Figure 2A and Figure S2F). However, *LKS1* expression did not completely disappear in the *stop1* mutant (Figure 3A), and the low expression level could still activate partial AKT1 channels (Figure 6A). Therefore, *stop1* mutant root could still respond to external LK and stop growth similar to wild type.

Both *LKS1 OE* and *STOP1 OE* showed an LK tolerant phenotype in shoot, which was due to increased K^+^ content in their shoot (Figure 3D,F). However, they displayed different root phenotypes. The *LKS1 OE* accumulated more K^+^ in root, which promoted root growth under LK conditions. On the other hand, the K^+^ content in *STOP1 OE* root was not significantly increased compared with wild type; therefore, *STOP1 OE* showed a similar root phenotype to wild type (Figure 3D,F). According to the root/shoot K^+^ distribution in *STOP1 OE* and *stop1/LKS1 OE* lines (Figure 3F and Figure 4E), STOP1 may also positively regulate some other genes involved in root-to-shoot K^+^ transport. In addition, both *lks1* mutant and *LKS1 OE* plants showed root growth phenotypes under LK conditions (Figure 2A and Figure 3D); however, the mechanisms should be different. As discussed above, loss of function of *LKS1* leads to a root growth phenotype under this LK condition. The complementation line (*lks1-3/pLKS1:VENUS-LKS1*) can restore this root phenotype to the wild-type level (Figure S8B). Comparatively, overexpression of *LKS1* enhanced K^+^ uptake and accumulation in root (Figure 3F), which led to the root growth phenotype in the *LKS1 OE* line. The *lks1/STOP1 OE* and *stop1/LKS1 OE* lines displayed *lks1* and *LKS1 OE* root phenotypes, respectively (Figure 4B,F). The *lks1 stop1* double mutant showed a root growth phenotype similar to the *lks1* mutant (Figure S5). All this genetic evidence and these root phenotypes indicated that *LKS1* could be the downstream target of STOP1.

**Figure 10:**
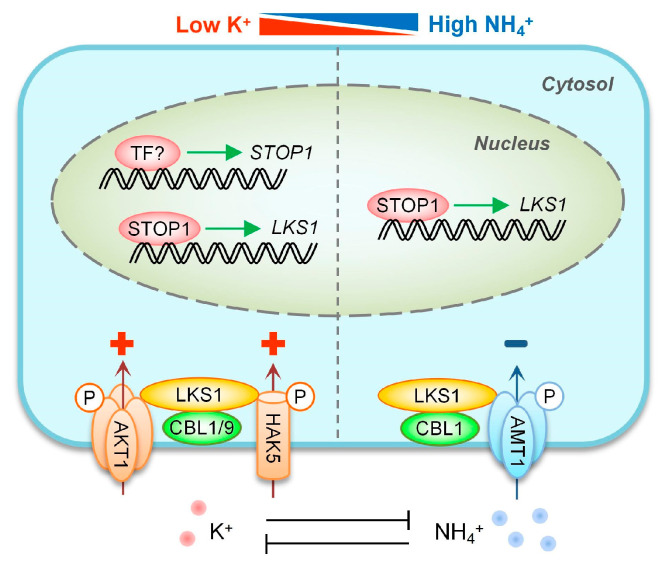
Working model of *LKS1* transcriptional regulation by STOP1 in *Arabidopsis* response to low-K^+^ and high-NH_4_^+^ stresses. Low K^+^, rather than high NH_4_^+^, can upregulate the transcript level of the *STOP1* gene. Both stresses result in STOP1 protein accumulation in the nucleus, where STOP1 activates *LKS1* transcription. Subsequently, LKS1 promotes AKT1-/HAK5-mediated K^+^ uptake and inhibits AMT1-mediated NH_4_^+^ uptake. The details of this schematic model are described in the text. “P” represents the phosphorylation process; “+” and “−” indicate positive and negative regulation, respectively.

In the present study, we identified an essential transcription factor STOP1 that is involved in *Arabidopsis* response to low-K^+^ and high-NH_4_^+^ stresses by regulating *LKS1* transcription. On the basis of previous studies, we extend the K^+^/NH_4_^+^ uptake regulatory network and propose a working model (Figure 10). When plants are subjected to low-K^+^ stress, the *STOP1* gene is somehow upregulated, and then STOP1 proteins directly bind to the *LKS1* promoter and induce *LKS1* transcription in the nucleus. Subsequently, LKS1 proteins are recruited to the PM by CBL1/CBL9 and phosphorylate the K^+^ channel AKT1 and the K^+^ transporter HAK5 to enhance K^+^ uptake in plant roots. In addition, high-NH_4_^+^ stress leads to STOP1 protein accumulation in nucleus, which positively regulates *LKS1* transcription. Then, the CBL1/LKS1 complex phosphorylates and represses the activities of NH_4_^+^ transporters AMTs to inhibit excess NH_4_^+^ uptake. Therefore, the STOP1/LKS1 pathway plays crucial roles in the K^+^ and NH_4_^+^ uptake/homeostasis, which coordinates potassium and nitrogen balance in plants responses to external fluctuating nutrient levels.

## 4. Materials and Methods

### 4.1. Plant Materials

The *Arabidopsis* (*Arabidopsis thaliana*) Columbia ecotype (Col-0) was used as the wild type in this study. The *Arabidopsis* mutants *stop1-2* (T-DNA insertion line, SALK_114108) and *stop1-1* (derived from an ethyl methanesulfonate-mutagenized M2 population of Col-0 in a previous study) [25] were obtained from the RIKEN Bio-Resource Center. The *lks1-3* (SALK_036154), *akt1* (SALK_071803), and *LKS1 OE* lines were obtained as described previously [11]. The *stop2* (SAIL_402_D03) and Col-3 lines were received from the Eurasian *Arabidopsis* Stock Center (uNASC). The *almt1* (SALK_009629) line was described previously [28]. *RAE1* overexpressing plants (*RAE1 OE-7* and *RAE1 OE-21*) were described previously [41].

The *STOP1* coding sequence was cloned into the pCAMBIA1300 vector (Cambia) driven by the *STOP1* native promoter (2 kb), and then the vector was transformed into the *stop1-2* mutant to obtain the complementation lines (*COM-1* and *COM-2*). The *pSuper:STOP1* vector was generated by cloning the *STOP1* CDS into pSuper1300 plasmid under the control of the *Super* promoter [44]. The *pSuper:STOP1* vector was transformed into Col-0 to acquire the *STOP1 OE* (*OE-1* and *OE-2*) transgenic plants. The *lks1-3*/*STOP1 OE* transgenic lines (*lks1-3*/*STOP1 OE-1* and *lks1-3*/*STOP1 OE-2*) were constructed by transforming the *pSuper:STOP1* vector into the *lks1-3* mutant. In the processes of vector construction, Phusion^®^ HF DNA Polymerase (M0530L, BioLabs) was used to clone the gene fragments, and the TAKARA DNA ligation system was used to obtain the genetic constructs. The recombinant plasmid was introduced into *E. coli* strain DH5α and the plasmid was extracted by AxyPrep^TM^ Plasmid Miniprep Kit (AXYGEN). The *stop1-1*/*LKS1 OE* line was generated by crossing *stop1-1* with *LKS1 OE*. To construct the *lks1 stop1-2* double mutants using the Crispr/Cas9 genome editing technique, two targets (Figure S5B) were designed within the *LKS1* genomic sequence on the website http://www.genome.arizona.edu/crispr/CRISPRsearch.html (10 December 2019), and the two targets were used to design the primers (LKS1 DT1-BsF, LKS1 DT2-BsR, LKS1 DT1-F0, LKS1 DT2-R0, as mentioned in Supplementary Table S1) for Crispr/Cas9 [45]. The *lks1 stop1-2* double mutants were produced by transforming the Crispr/Cas9 construct into *stop1-2* mutant. Genomic DNA from plants in the T_1_ generation was sequenced for construct verification. *Arabidopsis* transformation with *Agrobacterium* (strain GV3101) was carried out by the floral dip method [46].

Accession Numbers: Sequence data for the genes described in this article can be found in the *Arabidopsis* TAIR database (https://www.arabidopsis.org (3 January 2021)) under the following accession numbers: AT1G34370 for *STOP1*, AT1G30270 for *LKS1*/*CIPK23*, AT5G22890 for *STOP2*, AT2G26650 for *AKT1*, AT1G08430 for *ALMT1*, and AT1G80670 for *RAE1*.

### 4.2. Phenotypic Analyses and Growth Conditions

The *Arabidopsis* seeds were surface-sterilized using 6% (*v*/*v*) NaClO and incubated at 4 °C in darkness for 3 days. Then, the seeds were germinated on MS (Murashige & Skoog) medium at 22 °C under constant illumination at 60 μmol·m^–2^·s^–1^. All the medium used in this study contained 0.9% (*w*/*v*) agar (Ourchem) and 3% (*w*/*v*) sucrose (Sinopharm).

For the LK phenotype test, 5 day old seedlings grown on MS medium (20 mM K^+^) were transferred to LK medium (0.1 mM K^+^) or MS medium for 10 days (to observe the LK sensitive phenotype) or 12 days (to observe the LK tolerant phenotype). The LK medium was made by modifying the MS medium described previously [11]. The MS medium contained 1.5 mM MgSO_4_, 2.99 mM CaCl_2_, 20.6 mM NH_4_NO_3_, 18.79 mM KNO_3_, and 1.25 mM KH_2_PO_4_, while the LK medium contained 1.5 mM MgSO_4_, 2.99 mM CaCl_2_, 28.75 mM NH_4_NO_3_, and 1.25 mM NH_4_H_2_PO_4_. The final K^+^ concentration in the LK medium was adjusted to 0.1 mM by adding KCl. In the present study, LK referred to the low-K^+^ medium as described above, unless the concentrations were indicated.

For phenotypic assays on high-K^+^ (20 mM) and low-K^+^ (0.1 mM) medium in the absence of NH_4_^+^ (Figure 8), 5 day old seedlings grown on high-K^+^ (20 mM) medium were transferred to high-K^+^ (20 mM) and low-K^+^ (0.1 mM) medium for 10 days. The low-K^+^ (0.1 mM) medium contained 1.5 mM MgSO_4_, 1.25 mM H_3_PO_4_, 2.99 mM Ca(NO_3_)_2_, and 0.1 mM KCl. The high-K^+^ (20 mM) medium was supplemented with KCl to 20 mM.

For phenotypic assays on high-NH_4_^+^ (30 mM) and low-NH_4_^+^ (0 mM) medium (Figure 9), 5 day old seedlings grown on low-NH_4_^+^ (0 mM) medium were transferred to high-NH_4_^+^ (30 mM) and low-NH_4_^+^ (0 mM) medium for 10 days. The low-NH_4_^+^ (0 mM) medium contained 1.5 mM MgSO_4_, 1.25 mM H_3_PO_4_, 2.99 mM Ca(NO_3_)_2_, and 20 mM KCl. The high-NH_4_^+^ (30 mM) medium was supplemented with 30 mM NH_4_Cl. Other microelements in the above medium were consistent with MS medium.

For the phenotypic test, each plate contained four seedlings for each plant material. In one independent experiment, there were at least three biological replicates (three plates), and each phenotype test was performed at least three times.

For seed harvesting, *Arabidopsis* plants were cultured in the potting soil mixture (rich soil/vermiculite = 2:1, *v*/*v*) and kept in growth chambers (temperature was 22 °C, illumination was 120 μmol·m^−2^·s^−1^, and the relative humidity was approximately 70%) with long-day conditions (16 h light/8 h darkness).

### 4.3. K^+^ Content Measurement

The 5 day old *Arabidopsis* seedlings grown on MS medium were transferred to LK or MS medium and treated for the indicated times described in figure legends. For low-K^+^ treatment, 80 to 100 individual seedlings from two plates were collected as one biological replicate. For the K^+^ sufficient treatment, 40 to 50 individual seedlings from one plate were collected as one biological replicate. The shoots and roots were harvested separately. Three or four biological replicates (*n* = 3 or *n* = 4) were used in one independent experiment. To test the plants with the LK sensitive phenotype, K^+^ content was measured after 7 days of LK treatment. To test the plants with LK tolerant phenotype, K^+^ content was measured after 10 days of LK treatment.

To test the K^+^ content under low-K^+^ (0 mM NH_4_^+^) (Figure 8B) or high-NH_4_^+^ (Figure 9B) conditions, the 5 day old seedlings were transferred to low-K^+^ or high-NH_4_^+^ medium for 7 days. A total of 40 to 80 individual seedlings were collected as one biological replicate, and the shoots and roots were harvested separately. Three or four biological replicates (*n* = 3 or *n* = 4) were used in one independent experiment.

The collected samples were dried at 80 °C for 24 h to a constant weight, and then the dry weight was measured. The samples were treated in a muffle furnace at 300 °C for 1 h and then 575 °C for 5 h. The ashes were dissolved and diluted in 0.1 N HCl. The K^+^ concentrations were measured using the 4100-MP AES device (Agilent).

### 4.4. Transcription Analyses

For both RT-PCR and RT-qPCR analyses, total RNA was extracted from the roots of 7 day old seedlings using Trizol reagent (Invitrogen). The roots of 200 individual seedlings from two plates were collected and used as one biological replicate. Three or four biological replicates (*n* = 3 or *n* = 4) were used in one independent experiment. Then, 8 μg of total RNA was treated with DNase I (RNase Free, Takara) at 37 °C for 30 min and then 65 °C for 10 min to eliminate DNA contamination. Next, 4 μg of DNase I-treated RNA was used to synthesize complementary DNA (cDNA) with SuperScript^II^ RNase reverse transcriptase (Invitrogen). Oligo (dT) primers (Promega) were used for RT-PCR and Random Hexamer primers (Promega) were used for quantitative real-time PCR analyses (RT-qPCR).

For RT-PCR in Figure S2D, the *EF1α* gene was used as an internal standard for normalization of gene expression levels. The primers used to detect the CDS of *STOP1* are shown in Supplementary Table S1. PCR was performed for 30 cycles, each with 94 °C for 30 s, 60 °C for 30 s, and 72 °C for 1 min 50 s.

For RT-qPCR, the cDNA was diluted 40-fold with double-distilled water, and 8 μL of diluted cDNA was used as the template in each reaction. The Power SYBR Green PCR Master Mix (Applied Biosystems, USA) was used to carry out this assay. Herem 20 μL was one reaction volume containing 10 μL of SYBR Green premix, 8 μL of cDNA, and 2 μL of forward and reverse primers (1 μM), which was reacted on a 7500 Real Time PCR System machine (Applied Biosystems). The PCR was conducted as follows: 95 °C for 10 min, followed by 40 cycles of 95 °C for 15 s and 60 °C for 1 min. To normalize the test gene expression levels, *ACTIN2/8* was used as an internal standard. The primers used in this experiment are listed in Supplementary Table S1.

For the LK induction experiment in Figure 7A, 5 day old seedlings were transferred to MS or LK medium for 1 day. For RT-qPCR analyses of *STOP1* and *LKS1* expression in low-K^+^ response in the absence of NH_4_^+^ (Figure 8C), 5 day old seedlings were transferred to high-K^+^ (20 mM) and low-K^+^ (0.1 mM) medium for the indicated times. For RT-qPCR analyses of *STOP1* and *LKS1* expression in the high NH_4_^+^ response (Figure 9C), 5 day old seedlings were transferred to high-NH_4_^+^ (30 mM) and low-NH_4_^+^ (0 mM) medium for the indicated times. The roots of 200–240 individual seedlings were collected and used as one biological replicate for RT-qPCR assays. Three or four biological replicates (*n* = 3 or *n* = 4) were used in one independent experiment.

### 4.5. Mating-Based Y1H Screening

The *LKS1* promoter region was divided into four fragments, F1 (−382 to −1 bp), F2 (−757 to −364 bp), F3 (−1078 to −738 bp), and F4 (−1506 to −1061 bp), and the four fragments were constructed into the pHISi-1 vector, used as baits. For the mating-based Y1H screening, all the GAL4-AD-TF strains were grown overnight in SD/-Trp medium in 2 mL 96-well plates. Yeast strains YM4271 carrying the baits were grown overnight at the same time. Then, 20 μL of donor and host strains were transferred to a new 2 mL 96-well plate with 100 μL of YPAD medium. Mating was carried out for 24 h by shaking at 30 °C. After dilution with 1.5 mL of water, the mating products were plated on SD/-Trp-His selective plates and incubated for 3 days at 30 °C. Then, 5 mM or 10 mM 3-amino-1, 2, 4-triazole (3-AT) was added to SD/-Trp-His selective plates (Sigma-Aldrich).

### 4.6. Vector Constructions and Yeast One-Hybrid Assays

The *pLKS1:LacZ* construct was generated by cloning the *LKS1* promoter fragment (1506 bp) into pLacZi2μ vector [47]. To generate various *LacZ* reporter genes driven by the sub-fragments of the *LKS1* promoter shown in Figure 1B, the promoter fragments were amplified by PCR using *pLKS1:LacZ* construct as the template. The respective pairs of primers are shown in Supplementary Table S1. In order to generate the four types of mutants in the F2-2 fragment shown in Figure 5G, the QuikChange Site-Directed Mutagenesis Kit (Agilent) was used to conduct point mutation PCR, and the F2-2 fragment fused with *LacZ* reporter gene vector was used as the template. The reaction volume and PCR program referred to the manufacturer’s instructions. To generate *AD-STOP1*, the *STOP1* CDS was amplified by PCR and then cloned into the pB42AD vector (Clontech). To construct *AD-STOP1^H266Y^* vector, the template *AD-STOP1* plasmid and the QuikChange Site-Directed Mutagenesis Kit (Agilent) were used to produce the single-base mutation. The *LKS1* promoter fragments were co-transformed separately with *AD-STOP1* into the yeast strain EGY48. The transformed strains were cultured on SD/-Trp-Ura plates and confirmed by PCR. Then, these transformants were grown on proper SD/-Trp-Ura plates containing X-gal (5-bromo-4-chloro-3-indolyl-β-d-galactopyranoside), 2% galactose, and 1% raffinose for blue color development. The yeast transformation assay was conducted as described in the Yeast Protocols Handbook (Clontech).

### 4.7. Microscopy Imaging

The *stop1-2*/*pSTOP1:GFP-STOP1* and *stop1-2*/*pUBQ:GFP-STOP1* [28] plants were used to observe the fluorescence of GFP-STOP1. The *lks1-3*/*pLKS1:VENUS-LKS1* plants was constructed by transforming the *pLKS1:VENUS-LKS1* vector into *lks1-3* mutant. To observe the fluorescence induced by LK stress, the 5 day old seedlings of *stop1-2*/*pSTOP1:GFP-STOP1*, *stop1-2*/*pUBQ:GFP-STOP1*, and *lks1-3*/*pLKS1:VENUS-LKS1* plants were transferred to MS or LK medium for 1 day. To observe the fluorescence under low-K^+^ or high-NH_4_^+^ treatment (Figure 8 and Figure 9), the 5 day old seedlings were transferred to low-K^+^ or high-NH_4_^+^ medium for the indicated times. Then, the seedlings were used for the fluorescence observation. To show the outline of the root cells, seedlings were dipped in 30 μM propidium iodine (Sigma-Aldrich) solution for 1 min at room temperature and rinsed twice with double-distilled water. Images were collected on a Zeiss LSM710 confocal microscope using a Plan Apochromat ×40/1.4 Oil DIC M27 objective. GFP-STOP1 and PI were excited sequentially with a blue argon ion laser (488 nm, 45% strength) and a DPSS laser (561 nm, 1% strength). Emitted light was collected from 493 to 556 nm for GFP-STOP1 and from 647 to 721 nm for PI. Venus-LKS1 was excited with the blue argon ion laser (488 nm, 60% strength), and the emitted light was collected from 493 to 556 nm. The fluorescence intensity was measured by the Image J program. The photos of the experimental group and control group were taken with the same microscope and camera settings.

### 4.8. GUS/LUC Assay

*pLKS1:GUS* was constructed by cloning the *LKS1* promoter fragment (1.5 kb) with a *β-glucuronidase* (*GUS*) coding sequence into pCAMBIA1381 (Cambia) vector. The *pSuper:STOP1* and *pLKS1:GUS* vector were transfected into *Agrobacterium* (strain GV3101). *Agrobacterium* cells were harvested by centrifugation and suspended in the solutions containing 10 mM MES pH 5.6, 10 mM MgCl_2_, and 200 μM acetosyringone to an optical density (OD_600_) of 0.8, incubated at 28 °C for 2 h, and then the bacterial solution was injected into the *Nicotiana benthamiana* leaves. The bacterial solution was mixed in the following proportions: 300 μL of P19, 400 μL of *pLKS1:GUS*, 500 μL of *pSuper:STOP1*, and 4 μL of *pSuper:LUC* (*luciferase*). In the control group, *pSuper:STOP1* was replaced by *Super1300*. The GUS and LUC activity was measured after 3 days of injection. The GUS activity was measured using methyl umbelliferyl glucuronide (Sigma-Aldrich) and an F-4500 Flourescence Spectrophotometer (Hitachi). LUC activity was used as an internal control and measured by GloMax^®^ 20/20 Luminometer (Promega). The GUS/LUC ratio was used to determine the STOP1 binding activity to the *LKS1* promoter.

### 4.9. GUS Staining Assay

The *pLKS1:GUS* or *pSTOP1:GUS* vectors were generated by fusing the *LKS1* promoter fragment (1.5 kb) or *STOP1* promoter fragment (2 kb) with the *β-glucuronidase* (*GUS*) coding sequence into pCAMBIA1381 (Cambia). Then, the *pLKS1:GUS* vector was transformed into Col-0 and *stop1-1* to obtain the *pLKS1:GUS* and *stop1-1*/*pLKS1:GUS* transgenic plants. *pSTOP1:GUS* was obtained by transforming the *pSTOP1:GUS* vector into Col-0. For GUS staining assays, *pSTOP1:GUS* was incubated in GUS staining buffer for 15 min, and *pLKS1:GUS* or *stop1-1*/*pLKS1:GUS* was stained for 50 min at 37 °C. Here, 10 mL of GUS staining buffer contained 5 mg of X-gluc (BIOSYNTH, B-7300), 10–20 μL of *N*,*N*-dimethylformamide, 7.98 mL of PBS buffer (0.1 M, KH_2_PO_4_ and K_2_HPO_4_ with pH of 7.0), 1 mL of 5 mM potassium hexacyanoferrate (III), 1 mL of 5 mM potassium hexacyanoferrate (II), and 10 μL of Triton X-100.

### 4.10. ChIP-qPCR Assay

To construct *pSuper:STOP1-MYC*, the *STOP1* coding sequence fused with the MYC label was driven by the *Super* promoter and then transformed into Col-0. The *pSuper:STOP1-MYC* transgenic plant used in the ChIP assay was a single-copy homozygous line. The roots of 12 day old *pSuper:STOP1-MYC* seedlings (about 0.5–1 g fresh weight) were harvested and crosslinked with 1% formaldehyde for the ChIP experiment. Then, the nuclei were isolated, and the extracted nuclei were lysed according to [48] with minor modifications in lysis buffer composition (50 mM HEPES pH 7.5, 150 mM NaCl, 1 mM EDTA, 1 mM PMSF, 0.1% SDS, 0.1% Na deoxycholate, 1% Triton X-100, 1 μg·mL^−1^ pepstain A, 1 μg·mL^−1^ aprotinin). After the DNA was sheared, the sample was incubated with anti-MYC antibody (Abmart) to immunoprecipitate protein/DNA complexes. After reverse crosslinking and protein digestion, the precipitated DNA was used for qPCR detection. The primers are listed in Supplementary Table S1. qPCR was performed for 40 cycles, each at 95 °C for 15 s, 55 °C for 20 s, and 60 °C for 45 s. The detailed experimental methods were described previously [48].

### 4.11. EMSA (Electrophoretic Mobility Shift Assays)

The *STOP1* and *STOP1^C796T^* coding sequence was cloned into the pET-30a (+) vector (Novagen) to obtain the STOP1 and STOP1^H266Y^ protein expression plasmid. The recombinant plasmid was introduced into *E. coli* strain BL21. *E. coli* cells were induced with 0.2 mM IPTG overnight at 18 °C and collected by centrifugation (3220× *g*) at 4 °C for 10 min (Eppendorf centrifuge 5810 R with A-4-62 rotor). After the bacterial precipitation was cleaned once with His-Binding Buffer (20 mM Tris-HCl pH 8.0, 500 mM NaCl, 30 mM Imidazole), the precipitation was suspended with 10 mL of His-Binding Buffer (20 mM Tris-HCl pH 8.0, 500 mM NaCl, 30 mM Imidazole, 1 mM PMSF, 1 mM DTT), and the ultrasonication was performed on the bacterium suspension. A total of 99 ultrasounds were performed using 200 W of power for a 2 s ultrasound time and a 4 s interval. The ultrasonic solution was centrifuged (18,514× *g*) at 4 °C for 30 min (Eppendorf centrifuge 5810 R with F-34-6-38 rotor), and the supernatant of the bacteria lysate was used for protein purification. The protein was purified using Ni-Sepharose 6 Fast Flow (GE Healthcare). The wash buffer (20 mM Tris-HCl pH 8.0, 500 mM NaCl, 50 mM Imidazole) was used to wash Ni-Sepharose, and eluting buffer (20 mM Tris-HCl pH 8.0, 500 mM NaCl, 250 mM Imidazole) was used to elute protein. The protein concentration was determined by Bio-Rad protein assay and 250 μg of purified protein was used in EMSA experiment. The EMSA was conducted using LightShift Chemiluminescent EMSA Kit (ThermoFisher) according to the manufacturer’s protocol. The probes of the *LKS1* promoter were obtained by PCR using biotin-labeled or biotin-unlabeled primers. Biotin-unlabeled probes of the same sequences were used as competitors and His protein was used as the negative control.

### 4.12. Patch-Clamp Whole-Cell Recording from Root-Cell Protoplasts

Root-cell protoplasts were isolated by enzyme solution from 5 day old primary roots of *Arabidopsis* seedlings. The enzyme solution containing 1.5% (*w*/*v*) cellulysin (Calbiochem), 1.5% (*w*/*v*) cellulase RS (Yakult Honsha Co.), 0.1% (*w*/*v*) pectolyase Y-23 (Seishin Pharmaceutical Co.), and 0.1% (*w*/*v*) BSA was dissolved in standard solution containing 10 mM K^+^ glutamate, 2 mM MgCl_2_, 1 mM CaCl_2_, 350 mM sorbitol, and 5 mM MES (pH 5.8 adjusted with Tris). The primary roots were cut into small pieces and incubated in the enzyme solution at 23 °C for 40 min to release root-cell protoplasts. The protoplasts were filtered through 80 μm nylon mesh and washed twice with standard solution by centrifugation at 160× *g* for 5 min. The isolated root-cell protoplasts were kept on ice before patch-clamp experiments. The patch-clamp experiments were recorded using an Axopatch 200B amplifier (Axon Instruments) at room temperature in dim light. The contents of the bath and pipette solutions were the same as described previously [11].

### 4.13. Kinetic Analysis of K^+^ Uptake

*Arabidopsis* seeds were germinated on MS medium at 22 °C under constant illumination. For K^+^ depletion experiments, 6 day old seedlings were collected (0.6 g of fresh weight used as one biological replicate) and pretreated in one-quarter-strength MS solution (5.15 mM NH_4_NO_3_, 0.375 mM MgSO_4_, 4.7 mM KNO_3_, 0.31 mM KH_2_PO_4_, 0.75 mM CaCl_2_, and 5 mM MES, pH 5.8 adjusted with Tris) at 22 °C overnight. Then, the seedlings were transferred into 25 mL of K starvation solution (200 μM CaSO_4_, 5 mM MES, pH 5.8 adjusted with Tris) for 2 days. During these 2 days, K starvation solution was changed three times a day. Next, the seedlings were transferred to 25 mL of K depletion solution (200 μM CaSO_4_, 250 μM KNO_3_, 5 mM MES, pH 5.8 adjusted with Tris). All samples were shaken on a shaking table at 22 °C under constant illumination during the experiments [11]. The solution samples were collected at different timepoints indicated in Figure 6. The K^+^ concentrations were measured using the 4100-MP AES device (Agilent).

### 4.14. Statistical Analyses

Data were shown as means ± SE. Student’s *t*-test was used to analyze statistical significance between treatment and control. The *p*-value was shown as * *p* < 0.05 or ** *p* < 0.01 to indicate significant differences.

## Figures and Tables

**Table 1:** Statistical analysis of K^+^ currents recorded in the root cell protoplasts.

Current Density at −180 mV (pA/pF)	Col (*n* = 61)	*stop1-2* (*n* = 51)	*lks1-3* (*n* = 51)	*akt1* (*n* = 24)
0	0	0	9.8%	79.2%
0–50	14.8%	31.4%	88.2%	20.8%
50–150	29.5%	45.1%	2.0%	0
150–250	34.4%	15.7%	0	0
>250	21.3%	7.8%	0	0

## Data Availability

Data supporting the reported results may be supplied upon request by authors.

## Supplementary Materials

The following supporting information can be downloaded at: www.mdpi.com/article/10.3390/ijms23010383/s1.

## References

[1] Sodek L., Lea P.J., Miflin B.J. (1980). Distribution and Properties of a Potassium-dependent Asparaginase Isolated from Developing Seeds of Pisum sativum and Other Plants. Plant Physiol..

[2] Clarkson D.T., Hanson J.B. (1980). The Mineral Nutrition of Higher Plants. Ann. Rev. Plant Physiol..

[3] Leigh R.A., Wyn Jones R.G. (1984). A hypothesis relating critical potassium concentrations for growth to the distribution and functions of this ion in the plant-cell. New Phytol..

[4] Maathuis F.J. (2009). Physiological functions of mineral macronutrients. Curr. Opin. Plant. Biol..

[5] Schroeder J.I., Ward J.M., Gassmann W. (1994). Perspectives on the physiology and structure of inward-rectifying K^+^ channels in higher plants: Biophysical implications for K^+^ uptake. Annu. Rev. Biophys. Biomol. Struct..

[6] Mengel K., Kirkby E.A. (2001). Principles of Plant Nutrition.

[7] Zörb C., Senbayram M., Peiter E. (2014). Potassium in agriculture—Status and perspectives. J. Plant Physiol..

[8] Pettigrew W.T. (2008). Potassium influences on yield and quality production for maize, wheat, soybean and cotton. Physiol. Plant..

[9] Hirsch R.E., Lewis B.D., Spalding E.P., Sussman M.R. (1998). A role for the AKT1 potassium channel in plant nutrition. Science.

[10] Spalding E.P., Hirsch R.E., Lewis D.R., Qi Z., Sussman M.R., Lewis B.D. (1999). Potassium uptake supporting plant growth in the absence of AKT1 channel activity: Inhibition by ammonium and stimulation by sodium. J. Gen. Physiol..

[11] Xu J., Li H.D., Chen L.Q., Wang Y., Liu L.L., He L., Wu W.H. (2006). A protein kinase, interacting with two calcineurin B-like proteins, regulates K^+^ transporter AKT1 in *Arabidopsis*. Cell.

[12] Gierth M., Mäser P., Schroeder J.I. (2005). The potassium transporter AtHAK5 functions in K^+^ deprivation-induced high-affinity K^+^ uptake and AKT1 K^+^ channel contribution to K^+^ uptake kinetics in *Arabidopsis* roots. Plant Physiol..

[13] Pyo Y.J., Gierth M., Schroeder J.I., Cho M.H. (2010). High-affinity K^+^ transport in *Arabidopsis*: AtHAK5 and AKT1 are vital for seedling establishment and postgermination growth under low-potassium conditions. Plant Physiol..

[14] Li J., Long Y., Qi G.N., Li J., Xu Z.J., Wu W.H., Wang Y. (2014). The Os-AKT1 channel is critical for K^+^ uptake in rice roots and is modulated by the rice CBL1-CIPK23 complex. Plant Cell.

[15] Yang T., Zhang S., Hu Y., Wu F., Hu Q., Chen G., Cai J., Wu T., Moran N., Yu L. (2014). The role of a potassium transporter OsHAK5 in potassium acquisition and transport from roots to shoots in rice at low potassium supply levels. Plant Physiol..

[16] Qin Y.J., Wu W.H., Wang Y. (2019). ZmHAK5 and ZmHAK1 function in K^+^ uptake and distribution in maize under low K^+^ conditions. J. Integr. Plant Biol..

[17] Wang M.Y., Siddiqi M.Y., Glass A.D.M. (1996). Interactions between K^+^ and NH_4_^+^: Effects on ion uptake by rice roots. Plant Cell Environ..

[18] ten Hoopen F., Cuin T.A., Pedas P., Hegelund J.N., Shabala S., Schjoerring J.K., Jahn T.P. (2010). Competition between uptake of ammonium and potassium in barley and *Arabidopsis* roots: Molecular mechanisms and physiological consequences. J. Exp. Bot..

[19] Qi Z., Hampton C.R., Shin R., Barkla B.J., White P.J., Schachtman D.P. (2008). The high affinity K^+^ transporter AtHAK5 plays a physiological role in planta at very low K^+^ concentrations and provides a caesium uptake pathway in *Arabidopsis*. J. Exp. Bot..

[20] Rubio F., Nieves-Cordones M., Alemán F., Martínez V. (2008). Relative contribution of AtHAK5 and AtAKT1 to K^+^ uptake in the high-affinity range of concentrations. Physiol. Plant..

[21] Ragel P., Ródenas R., García-Martín E., Andrés Z., Villalta I., Nieves-Cordones M., Rivero R.M., Martínez V., Pardo J.M., Quintero F.J. (2015). The CBL-Interacting Protein Kinase CIPK23 Regulates HAK5-Mediated High-Affinity K^+^ Uptake in *Arabidopsis* Roots. Plant Physiol..

[22] Scherzer S., Böhm J., Krol E., Shabala L., Kreuzer I., Larisch C., Bemm F., Al-Rasheid K.A., Shabala S., Rennenberg H. (2015). Calcium sensor kinase activates potassium uptake systems in gland cells of Venus flytraps. Proc. Natl. Acad. Sci. USA.

[23] Ho C.H., Lin S.H., Hu H.C., Tsay Y.F. (2009). CHL1 functions as a nitrate sensor in plants. Cell.

[24] Straub T., Ludewig U., Neuhäuser B. (2017). The Kinase CIPK23 Inhibits Ammonium Transport in *Arabidopsis thaliana*. Plant Cell.

[25] Iuchi S., Koyama H., Iuchi A., Kobayashi Y., Kitabayashi S., Kobayashi Y., Ikka T., Hirayama T., Shinozaki K., Kobayashi M. (2007). Zinc finger protein STOP1 is critical for proton tolerance in *Arabidopsis* and coregulates a key gene in aluminum tolerance. Proc. Natl. Acad. Sci. USA.

[26] Liu J.P., Magalhaes J.V., Shaff J., Kochian L.V. (2009). Aluminum-activated citrate and malate transporters from the MATE and ALMT families function independently to confer *Arabidopsis aluminum* tolerance. Plant J..

[27] Sawaki Y., Iuchi S., Kobayashi Y., Kobayashi Y., Ikka T., Sakurai N., Fujita M., Shinozaki K., Shibata D., Kobayashi M. (2009). STOP1 regulates multiple genes that protect arabidopsis from proton and aluminum toxicities. Plant Physiol..

[28] Balzergue C., Dartevelle T., Godon C., Laugier E., Meisrimler C., Teulon J.M., Creff A., Bissler M., Brouchoud C., Hagège A. (2017). Low phosphate activates STOP1-ALMT1 to rapidly inhibit root cell elongation. Nat. Commun..

[29] Mora-Macías J., Ojeda-Rivera J.O., Gutiérrez-Alanís D., Yong-Villalobos L., Oropeza-Aburto A., Raya-González J., Jiménez-Domínguez G., Chávez-Calvillo G., Rellán-Álvarez R., Herrera-Estrella L. (2017). Malate-dependent Fe accumulation is a critical checkpoint in the root developmental response to low phosphate. Proc. Natl. Acad. Sci. USA.

[30] Yamaji N., Huang C.F., Nagao S., Yano M., Sato Y., Nagamura Y., Ma J.F. (2009). A zinc finger transcription factor ART1 regulates multiple genes implicated in aluminum tolerance in rice. Plant Cell.

[31] Sawaki Y., Kobayashi Y., Kihara-Doi T., Nishikubo N., Kawazu T., Kobayashi M., Kobayashi Y., Iuchi S., Koyama H., Sato S. (2014). Identification of a STOP1-like protein in Eucalyptus that regulates transcription of Al tolerance genes. Plant Sci..

[32] Yokosho K., Yamaji N., Ma J.F. (2014). Global transcriptome analysis of Al-induced genes in an Al-accumulating species, common buckwheat (*Fagopyrum esculentum* Moench). Plant Cell Physiol..

[33] Fan W., Lou H.Q., Gong Y.L., Liu M.Y., Cao M.J., Liu Y., Yang J.L., Zheng S.J. (2015). Characterization of an inducible C2H2 -type zinc finger transcription factor VuSTOP1 in rice bean (*Vigna umbellata*) reveals differential regulation between low pH and aluminum tolerance mechanisms. New Phytol..

[34] Ohyama Y., Ito H., Kobayashi Y., Ikka T., Morita A., Kobayashi M., Imaizumi R., Aoki T., Komatsu K., Sakata Y. (2013). Characterization of *AtSTOP1* orthologous genes in tobacco and other plant species. Plant Physiol..

[35] Fan W., Lou H.Q., Yang J.L., Zheng S.J. (2016). The roles of STOP1-like transcription factors in aluminum and proton tolerance. Plant Signal Behav..

[36] Ou B., Yin K.Q., Liu S.N., Yang Y., Gu T., Hui J.M.W., Zhang L., Miao J., Kondou Y., Matsui M. (2011). A high-throughput screening system for *Arabidopsis* transcription factors and its application to Med25-dependent transcriptional regulation. Mol. Plant.

[37] Liu K.H., Huang C.Y., Tsay Y.F. (1999). CHL1 is a dual-affinity nitrate transporter of *Arabidopsis* involved in multiple phases of nitrate uptake. Plant Cell.

[38] Wang R., Liu D., Crawford N.M. (1998). The *Arabidopsis* CHL1 protein plays a major role in high-affinity nitrate uptake. Proc. Natl. Acad. Sci. USA.

[39] Godon C., Mercier C., Wang X., David P., Richaud P., Nussaume L., Liu D., Desnos T. (2019). Under phosphate starvation conditions, Fe and Al trigger accumulation of the transcription factor STOP1 in the nucleus of *Arabidopsis* root cells. Plant J..

[40] Sadhukhan A., Enomoto T., Kobayashi Y., Watanabe T., Iuchi S., Kobayashi M., Sahoo L., Yamamoto Y.Y., Koyama H. (2019). Sensitive to Proton Rhizotoxicity1 Regulates Salt and Drought Tolerance of *Arabidopsis thaliana* through Transcriptional Regulation of *CIPK23*. Plant Cell Physiol..

[41] Zhang Y., Zhang J., Guo J., Zhou F., Singh S., Xu X., Xie Q., Yang Z., Huang C.F. (2019). F-box protein RAE1 regulates the stability of the aluminum-resistance transcription factor STOP1 in *Arabidopsis*. Proc. Natl. Acad. Sci. USA.

[42] Tokizawa M., Kobayashi Y., Saito T., Kobayashi M., Iuchi S., Nomoto M., Tada Y., Yamamoto Y.Y., Koyama H. (2015). Sensitive to proton rhizotoxicity1, calmodulin binding transcription activator2, and other transcription factors are involved in *aluminum-activated malate transporter1* expression. Plant Physiol..

[43] Li J., Wu W.H., Wang Y. (2017). Potassium channel AKT1 is involved in the auxin-mediated root growth inhibition in *Arabidopsis* response to low K^+^ stress. J. Integr. Plant Biol..

[44] Ni M., Cui D., Einstein J., Narasimhulu S., Vergara C.E., Gelvin S.B. (1995). Strength and tissue specificity of chimeric promoters derived from the octopine and mannopine synthase genes. Plant J..

[45] Xing H.L., Dong L., Wang Z.P., Zhang H.Y., Han C.Y., Liu B., Wang X.C., Chen Q.J. (2014). A CRISPR/Cas9 toolkit for multiplex genome editing in plants. BMC Plant Biol..

[46] Clough S.J., Bent A.F. (1998). Floral dip: A simplified method for *Agrobacterium*-mediated transformation of *Arabidopsis thaliana*. Plant J..

[47] Lin R., Ding L., Casola C., Ripoll D.R., Feschotte C., Wang H. (2007). Transposase-derived transcription factors regulate light signaling in *Arabidopsis*. Science.

[48] Saleh A., Alvarez-Venegas R., Avramova Z. (2008). An efficient chromatin immunoprecipitation (ChIP) protocol for studying histone modifications in *Arabidopsis* plants. Nat. Protoc..

