# Supplementary material for: STOP1 Regulates LKS1 Transcription and Coordinates K+/NH4+ Balance in Arabidopsis Response to Low-K+ Stress
Source: Int J Mol Sci. 2021 Dec 29;23(1):383. doi: 10.3390/ijms23010383 (PMC8745191; doi:10.3390/ijms23010383)
Supplement: Supplementary file 1 [file ijms-23-00383-s001.zip › ijms-1504682-supplementary.pdf]

Figure S1

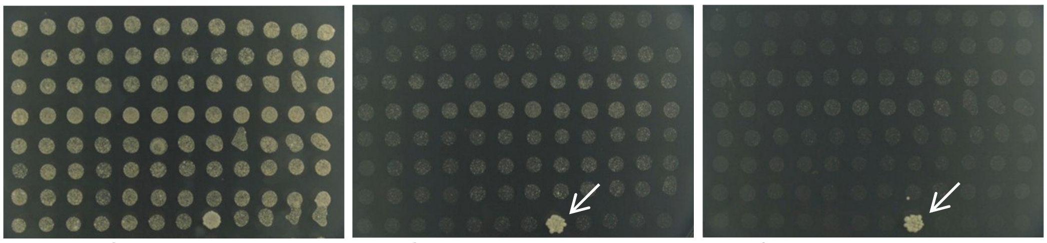

Figure S1. Isolation of the transcription factors of *LKS1* using high-throughput yeast-one-hybrid screening method (Supports Figure 1).

The F2 fragment (-757 to -364 bp) of *LKS1* promoter was used as a bait in yeast-one-hybrid screening. Each spot represents a transcription factor, the arrow indicates STOP1. The interaction between STOP1 and F2 fragment could be detected on the medium SD/-Trp-His with 3-amino-1,2,4-triazole (3-AT). Yeast grown on the medium SD/-Trp-His without 3-AT was shown to indicate mating efficiency.

Figure S2

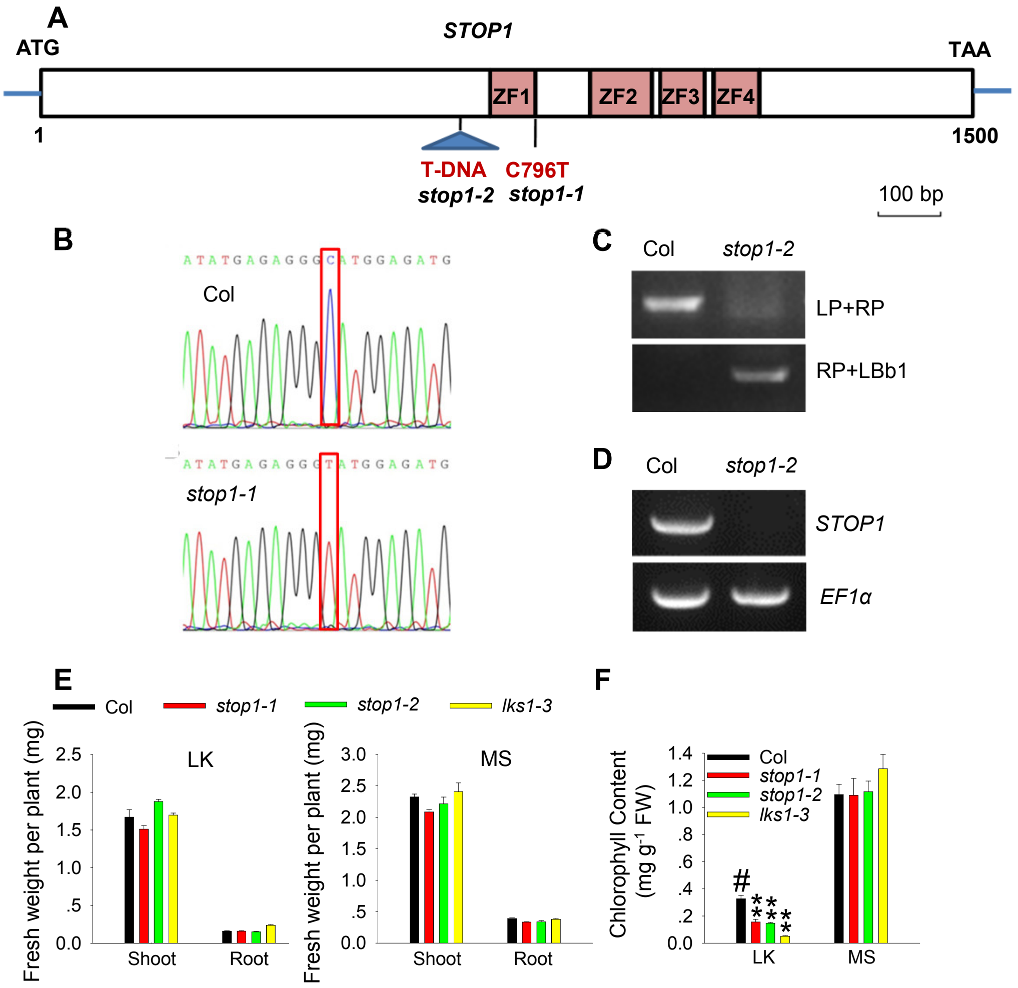

Figure S2. Molecular verification and growth data of *stop1-1* and *stop1-2* mutants (Supports Figure 2).

(A) Schematic representation of *STOP1* gene structure. Red boxes indicate four zinc finger domains (ZF1 to ZF4). The mutation site in *stop1-1* (C796T) and T-DNA insertion site in *stop1-2* (SALK_114108) are indicated, respectively.

(B) Partial alignment of the *stop1-1* mutant sequence with the wild-type sequence. The red boxes indicate the location of C796T mutation in *stop1-1* mutant.

(C) T-DNA insertion verification of *stop1-2* mutant (SALK_114108).

(D) RT-PCR test of *STOP1* expression in wild type (Col) and *stop1-2* mutant.

(E) Fresh weight of Col, *stop1-1*, *stop1-2* and *lks1-3*. The 5-day-old seedlings were transferred to MS or LK medium for 7 d, and then the shoots and roots were collected to test the fresh weight. Data are shown as means ± SE (n = 4).

(F) Chlorophyll content of Col, *stop1-1*, *stop1-2* and *lks1-3*. The 5-day-old seedlings were transferred to MS or LK medium for 10 d, and then the shoot was collected to test the Chlorophyll content. Data are shown as means ± SE (n = 4). Student's *t* test (*P < 0.05 and **P < 0.01) was used to analyze statistical significance, and # represents control.

Figure S3

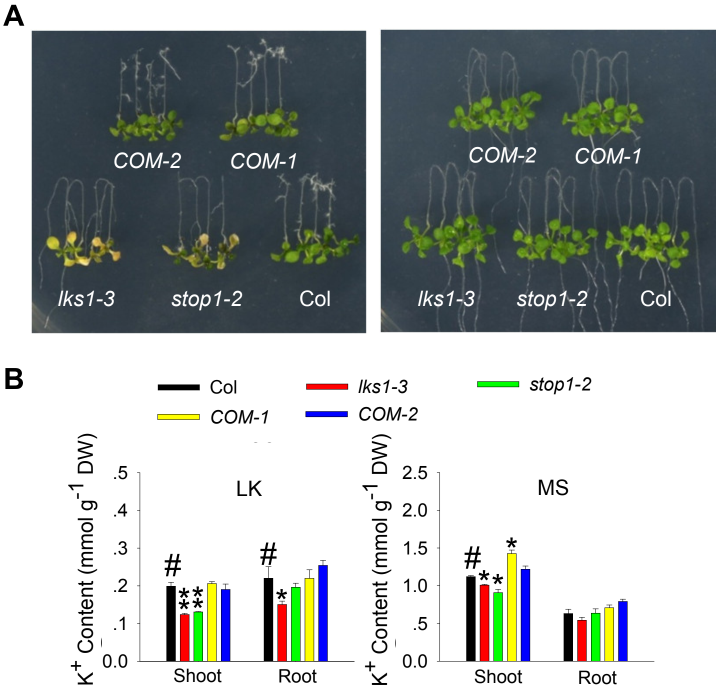

Figure S3. Phenotype test and K^+ content measurement of *stop1* complementation lines (Supports Figure 2).

(A) Phenotype test of *stop1* complementation lines (*COM-1* and *COM-2*). The 5-day-old seedlings were transferred to MS or LK medium for 10 d.

(B) K^+ content measurement of indicated plants shown in **(A)** after being transferred to MS or LK medium for 7 d. Data are shown as means ± SE (n = 3). Student's t test (*P < 0.05 and **P < 0.01) was used to analyze statistical significance, and # represents control.

Figure S4

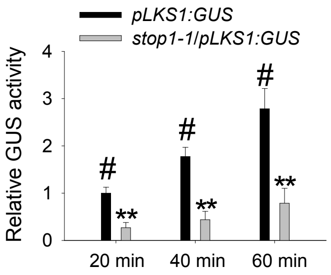

Figure S4. GUS activity measurement in *pLKS1:GUS* and *stop1-1/pLKS1:GUS* plants (Supports Figure 3B).

GUS activities were measured at the indicated reaction times. Data are shown as means ± SE (*n* = 4). Student's *t* test (*P < 0.05 and **P < 0.01) was used to analyze statistical significance, and # represents control.

Figure S5

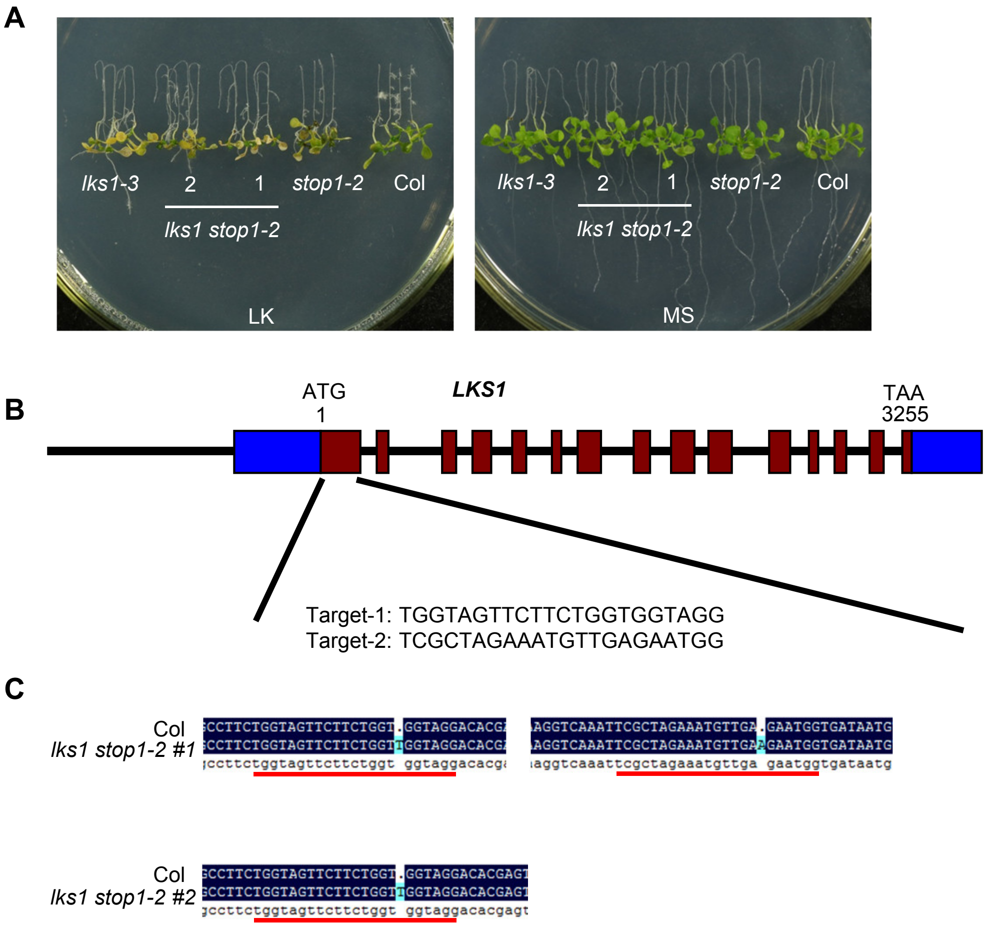

Figure S5. Phenotype test of *lks1 stop1* double mutants (Supports Figure 4).

(A) Phenotype comparison of Col, *stop1-2*, *lks1-3* and *lks1 stop1-2* double mutants. Seeds were germinated on MS medium for 5 d, then the seedlings were transferred to MS or LK medium for 10 d.

(B) Schematic representation of *lks1* gene structure and Crispr/Cas9 target position. Two target sequences are located on the first exon of *LKS1* gene. The *lks1 stop1-2* double mutants were constructed by knocking the *LKS1* gene in *stop1-2* mutant.

(C) The mutation form of the *LKS1* gene in *lks1 stop1-2* double mutants (#1 and #2). The mutation in *LKS1* was evaluated by sequencing. The red lines indicate the location of the targets. In #1 line, a T was inserted into the first target and an A was inserted into the second target. In #2 line, a T was inserted into the first target.

Figure S6

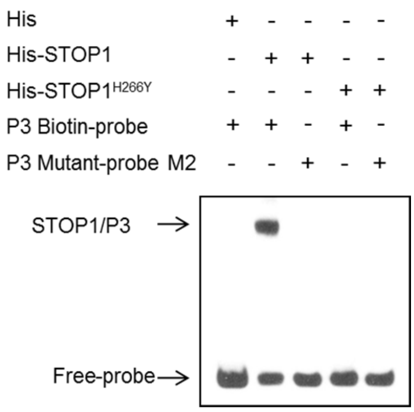

Figure S6. EMSA analyses showing the DNA binding activity of STOP1^{H266Y} (Supports Figure 5). The purified His-STOP1 and His-STOP1^{H266Y} proteins were incubated with P3 probes labeled with biotin. The P3 mutant probe M2 was used here as a negative control. The mutant probe M2 was shown in Figure 5G.

Figure S7

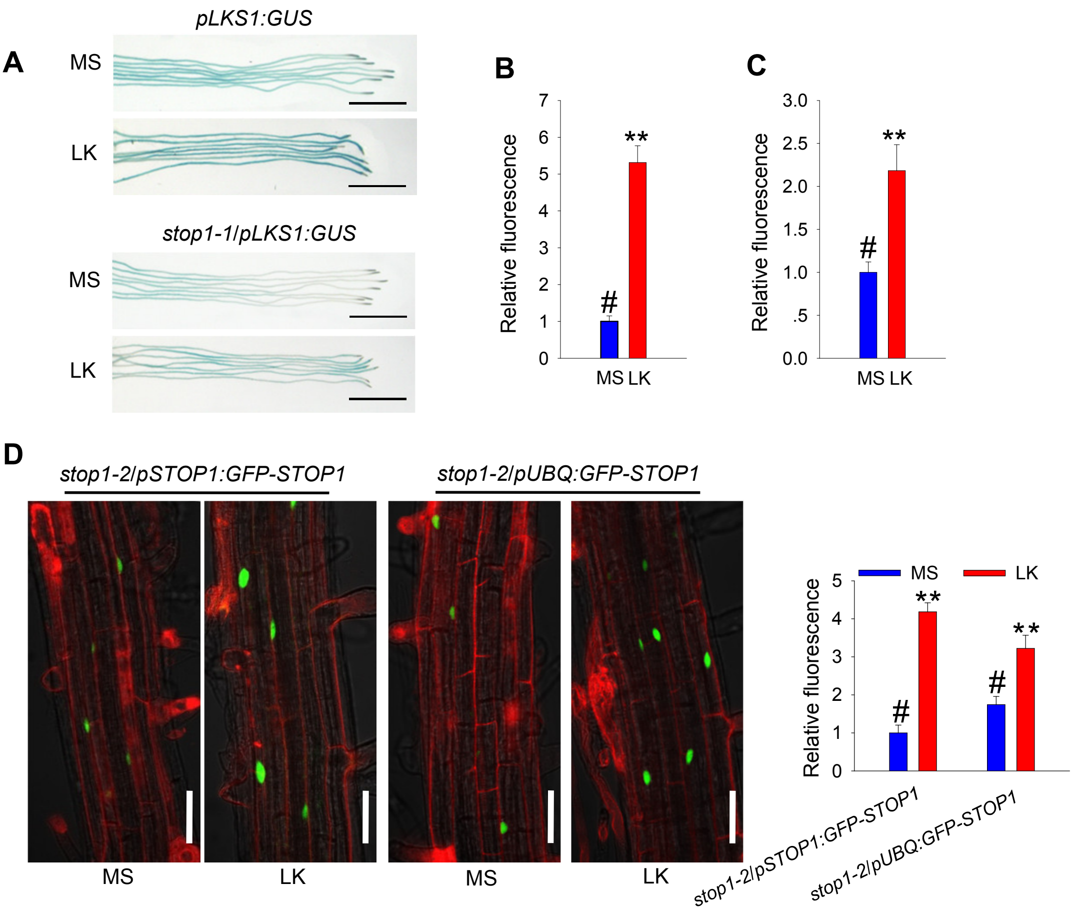

Figure S7. GUS staining assay and fluorescence observation (Supports Figure 7).

(A) GUS staining showing *LKS1* expression in wild-type (Col) and *stop1-1* background. The 5-day-old seedlings (seeds were germinated on MS medium) were transferred to MS or LK medium for 1 d, and then used for GUS staining. Scale bars =2 mm.

(B and C) Data in this figure support Figure 7C. The relative fluorescence intensity of GFP-STOP1 (B) (n=10) and VENUS-LKS1 (C) (n=5) were calculated. Data are shown as means ± SE. Student's *t* test (*P < 0.05 and **P < 0.01) was used to analyze statistical significance, and # represents control.

(D) Fluorescence observation showing the protein expression of GFP-STOP1. The red fluorescence was due to the propidium iodine staining. The 5-day-old seedlings were transferred to MS or LK medium for 1 d, and then used for fluorescence observation. Scale bars = 50 μm. The relative fluorescence intensity of GFP-STOP1 (n=12) were calculated. Data are shown as means ± SE. Student's *t* test (*P < 0.05 and **P < 0.01) was used to analyze statistical significance, and # represents control.

Figure S8

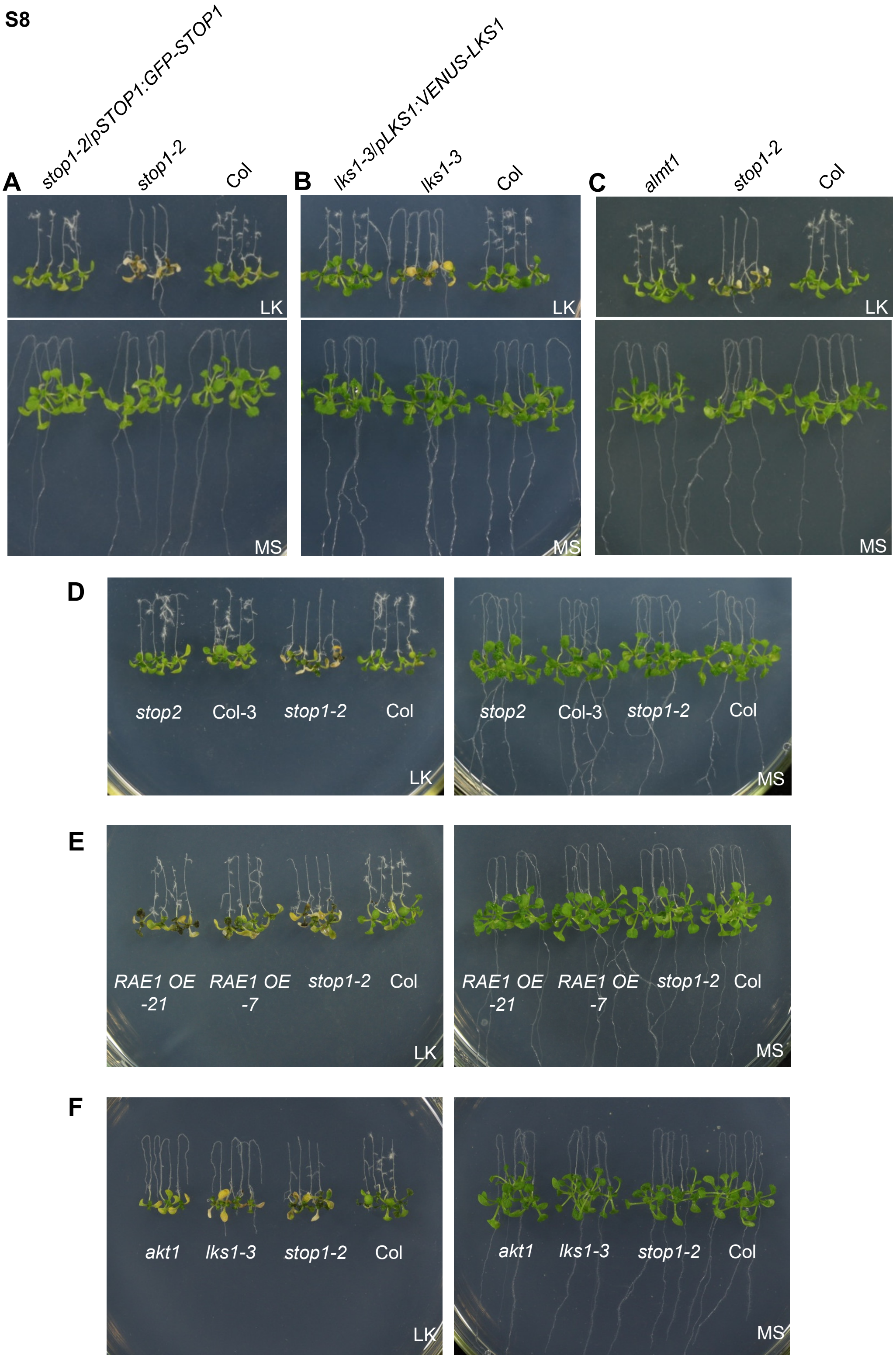

Figure S8. Phenotype test of seedlings under LK conditions (Supports Figure 7).

Phenotype test of *stop1-2/pSTOP1:GFP-STOP1* **(A)**, *lks1-3/pLKS1:VENUS-LKS1* **(B)**, *almt1* mutant **(C)**, *stop2* mutant **(D)**, RAE1 overexpressing line (*RAE1 OE-7* and *RAE1 OE-21*) **(E)** The 5-day-old seedlings were transferred to MS or LK medium for 10 d. **(F)** The 5-day-old seedlings were transferred to MS or LK medium for 10 d. The MS or LK medium contained 5 mM MES and adjusted pH to 5.8 with Tris.

Figure S9

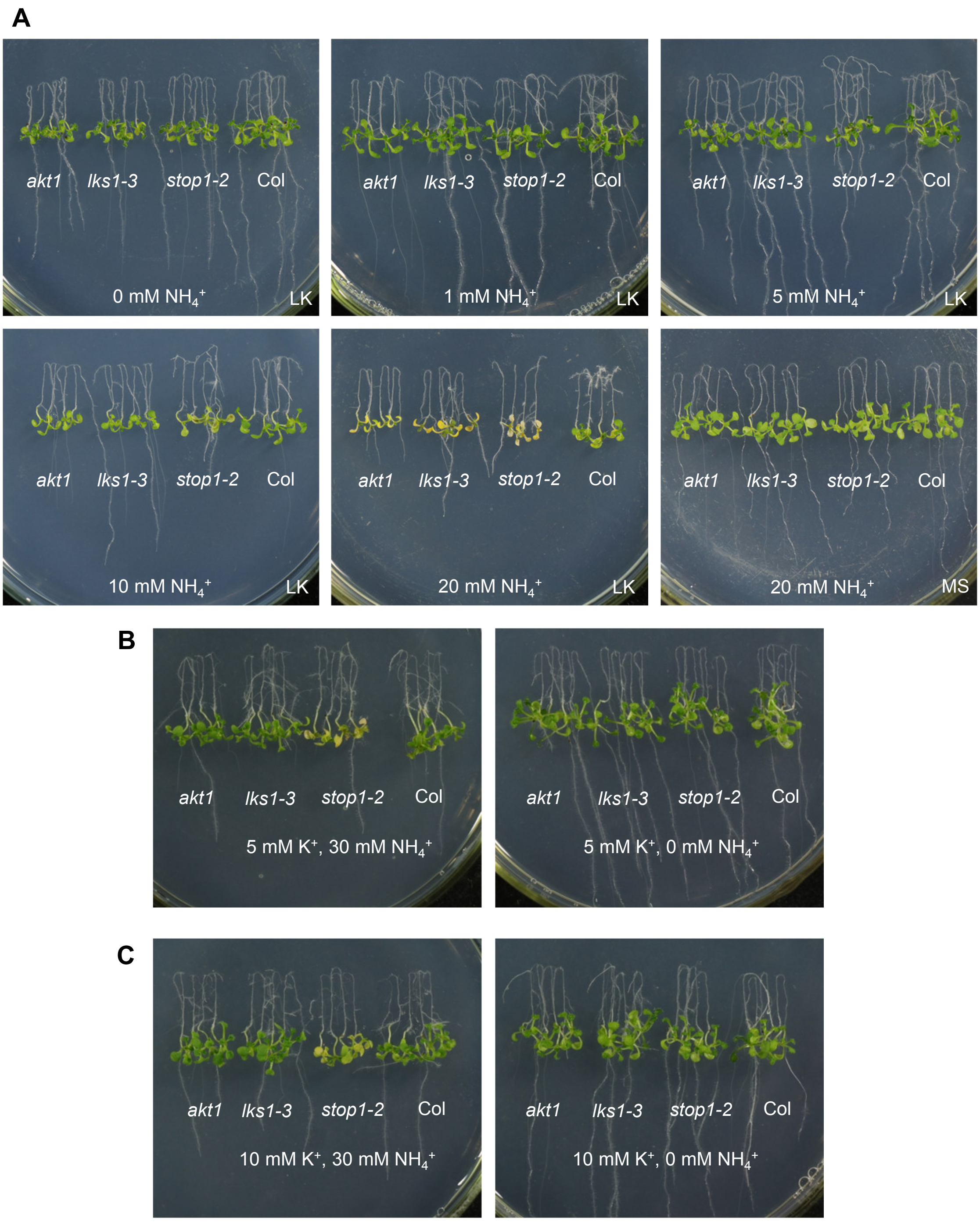

Figure S9. Phenotype test of seedlings under different NH_4^+ and K^+ conditions (Supports Figure 9).

(A) Seeds were germinated on MS medium for 5 d, then the seedlings were transferred to MS or the LK (0.1 mM K^+) medium containing different NH_4^+ concentrations for 10 d.

(B and C) Seeds were germinated on low NH_4^+ (0 mM) medium for 5 d, then the seedlings were transferred to high NH_4^+ (5 mM K^+, 30 mM NH_4^+ or 10 mM K^+, 30 mM NH_4^+) and low NH_4^+ medium for 10 d.

Supplemental Table S1. Primer Sequences Used in This Study.

Primer name	Sequence (5' to 3')	Note
**Primers for mutants identification and transgenic construction**		
stop1-2-LP<br>*stop1-2*-RP<br>LBb1	TTCATTGGTGAGAACGACTCC<br>ATCTTCTTGTTGGTCGTGGTG<br>GCGTGGACCGCTTGCTGCAACT	T-DNA identification
lks1-3-LP<br>*lks1-3*-RP	TTGTGATCCTCTTGCATAGGG<br>AATCATCCCGGACAAAGTACC	T-DNA identification
stop1-1-F<br>*stop1-1*-R	CTTATAACGACGGATCTTACTTCC<br>GTACCACAGGAACAAAGCCAC	*stop1-1* identification
STOP1 COM-F<br>STOP1 COM-R	AAGGTACCCTGTTGGCGAAGAAGTAACTAAGGGTTG<br>AATCTAGAGATACTGACAAGCTAAAGCACACTCTCGC	pCAMBIA1300 (Primers used to construct *STOP1 COM*)
STOP1-XbaI-F<br>STOP1-KpnI-R	AATCTAGAATGGAAACTGAAGACGATTTGTGC<br>AAGGTACCTTAGAGACTAGTATCTGAAACAGAC	pSuper1300<br>pSuper1300-MYC
STOP1-Pro-F<br>STOP1-Pro-R<br>LKS1-Pro-F<br>LKS1-Pro-R	AAGAATTCTAGGGCTTCAAACTCTTTACCATATAT<br>AAGTCGACTTTTTAGTTCAAGATCTTGTTTTTC<br>AAGAATTCCCTGGCGAGTATGTTCTCAC<br>TTGTCGACCTCTCTCTCTCTCGGTC	pCAMBIA1381 (Primers used to construct *pSTOP1:GUS* and *pLKS1:GUS*
**Primers used for RT-PCR**		
STOP1-RT PCR-F<br>STOP1-RT PCR-R	ATGGAAACTGAAGACGATTTGTGC<br>TTAGAGACTAGTATCTGAAACAGAC	
EF1α-F<br>EF1α-R	ATGCCCCAGGACATCGTGATTTCAT<br>TTGGCGGCACCCTTACGTGGATCA	
**Primers used for RT-qPCR**		
STOP1-qRT-F<br>STOP1-qRT-R	ATTTGCCTAAGCCGGTTCTT<br>ATGCCCTCTCATATGCATCC	
LKS1-qRT-F<br>LKS1-qRT-R	CAAGTTCGAGAGGATGGGTTAC<br>TCTTAGCACTTGCAGAGAACCA	
Actin2/8-qRT-F<br>Actin2/8-qRT-R	ACGGTAACATTGTGCTCAGTGGTG<br>CTTGGAGATCCACATCTGCTGGA	
**Primers used in yeast assay**		
LKS1 (-1~-382)-F<br>LKS1 (-1~-382)-R<br>LKS1 (-364~-757)-F<br>LKS1 (-364~-757)-R<br>LKS1 (-738~-1078)-F<br>LKS1 (-738~-1078)-R<br>LKS1 (-1061~-1506)-F<br>LKS1 (-1061~-1506)-R	AAGGTACCAATCACAATCTCTCTCTCTCTCG<br>AAGTCGACCTCTCTCTCTATATATATATCTCGG<br>AAGGTACCTTCTTTAACTCTTTTGAAATCGTTG<br>AAGTCGACAGAGAGAGAGAGATTGTGATTCGATG<br>AAGGTACCAAATAAAAGATCCTCTTACGTTTTA<br>AAGTCGACTTTCAAAAGAGTTAAAGAACGACAG<br>AAGGTACCCGTGGCAAGGTCATGTGTA<br>AAGTCGACAAGAGGATCTTTTATTTACTCTAATA	pLacZi2μ
LKS1 (-760~-641)-F<br>LKS1 (-760~-641)-R<br>LKS1 (-660~-541)-F<br>LKS1 (-660~-541)-R<br>LKS1 (-560~-441)-F<br>LKS1 (-560~-441)-R<br>LKS1 (-460~-361)-F<br>LKS1 (-460~-361)-R	TTGAATTCTCGTTCTTTAACTCTTTTGAAATCG<br>TATCTAGATTTACATGCCGTGTAATTAGCTGTC<br>AAGAATTCCTAATTACACGGCATGTAAAAAAG<br>ATTCTAGACTTTGATAATTTAAATGCAAACGTTA<br>AAGAATTCTTGCATTTAAATTATCAAAGACTAAG<br>TATCTAGAAATTAAAATTCTGTTTAGGGATTATAC<br>ATGAATTCCCCTAAACAGAATTTTAATTAAAC<br>AATCTAGAGAGAGAGAGAGAGATTGTGATTC	pLacZi2μ

Primer name	Sequence (5' to 3')	Note
**Primers used in yeast assay**		
LKS1 (-560~-441)-M1-F<br>LKS1 (-560~-441)-M1-R<br>LKS1 (-560~-441)-M2-F<br>LKS1 (-560~-441)-M2-R<br>LKS1 (-560~-441)-M3-F<br>LKS1 (-560~-441)-M3-R<br>LKS1 (-560~-441)-M4-F<br>LKS1 (-560~-441)-M4-R	ACGTCAGAACCTACAGATACGGTCTTCCTCGTATAATCCCTAAACAGAA<br>TTCTGTTTAGGGATTATACGAGGAAGACCGTATCTGTAGGTTCTGACGT<br>ACGTCAGAACCTACAGATACAACTGGCCTCGTATAATCCCTAAACAGAA<br>TTCTGTTTAGGGATTATACGAGGCCAGTTGTATCTGTAGGTTCTGACGT<br>TCAGAACCTACAGATACAACCTTTTGCGTATAATCCCTAAACAGAATTT<br>AAATTCTGTTTAGGGATTATACGCAAAAGGTTGTATCTGTAGGTTCTGA<br>GAACCTACAGATACAACCTTCCTTTGATAATCCCTAAACAGAATTTTAA<br>TTAAAATTCTGTTTAGGGATTATCAAAGGAAGGTTGTATCTGTAGGTTC	pLacZi2μ
STOP1-EcoRI-F<br>STOP1-XhoI-R	AAGAATTCATGGAAACTGAAGACGATTTGTGC<br>ATCTCGAGTTAGAGACTAGTATCTGAAACAG	pB42AD
**Primers used in EMSA**		
STOP1-pET30a-F<br>STOP1-pET30a-R	AAGATATCATGGAAACTGAAGACGATTTGTGC<br>ATGTCGACTTAGAGACTAGTATCTGAAACAG	pET30a (+)
LKS1 (-560~-441)-F<br>LKS1 (-560~-441)-R	TTGCATTTAAATTATCAAAGACTAAG<br>AATTAAAATTCTGTTTAGGGATTATAC	
**Primers used in ChIP-qPCR**		
LKS1 ChIP P1-F<br>LKS1 ChIP P1-R	CGTGGCAAGGTCATGTGTAT<br>CAACCCAAAATAGTTTAAACCTAG	
LKS1 ChIP P2-F<br>LKS1 ChIP P2-R	GTGGTGGACTTAGTTCAATATTTTT<br>TTCTTACCTAACTTAAATAATATTCTCG	
LKS1 ChIP P3-F<br>LKS1 ChIP P3-R	TTGCATTTAAATTATCAAAGACTAAG<br>AATTAAAATTCTGTTTAGGGATTATAC	
LKS1 ChIP P4-F<br>LKS1 ChIP P4-R	ACGAGAGTTGAATTTTGATTTTC<br>TCTATTGAATCAGGAGGAGGAC	
LKS1 ChIP G1-F<br>LKS1 ChIP G1-R	ATTTCCATGATTTATTCAAAAGC<br>TGTGTGTCAGTTTTCAGTAGCAG	
ChIP Actin-F<br>ChIP Actin-R	CCGGTATTGTGCTCGATTCTG<br>TTCCCGTTCTGCGGTAGTGG	
**Primers used in Crispr/Cas9**		
LKS1 DT1-BsF<br>LKS1 DT2-BsR<br>LKS1 DT1-F0<br>LKS1 DT2-R0	ATATATGGTCTCGATTGTGGTAGTTCTTCTGGTGGTGTT<br>ATTATTGGTCTCGAAACTTCTCAACATTTCTAGCGAC<br>TGTGGTAGTTCTTCTGGTGGTGTTTTAGAGCTAGAAATAGC<br>AACTTCTCAACATTTCTAGCGACAATCTCTTAGTCGACTCTAC	pHSE401
